# Oxidative Phosphorylation and Fatty Acid Oxidation Are Central to Mitochondrial Metabolism Rewiring in CML Stem/Progenitor Cell Survival

**DOI:** 10.3390/pathophysiology33030056

**Published:** 2026-08-06

**Authors:** Jelena Milenkovic, Dijana Stojanovic, Branka Djordjevic, Sanja Velickovic, Vladana Stojiljkovic, Milica Veljkovic, Maja Milojkovic

**Affiliations:** 1 Department of Pathophysiology, Faculty of Medicine, University of Nis, 18000 Nis, Serbia; dijana.stojanovic@medfak.ni.ac.rs (D.S.); maja.milojkovic@medfak.ni.ac.rs (M.M.); 2 Department of Biochemistry, Faculty of Medicine, University of Nis, 18000 Nis, Serbia; branka.djordjevic@medfak.ni.ac.rs (B.D.); vladana.stojiljkovic@medfak.ni.ac.rs (V.S.); 3 Department of Internal Medicine, Faculty of Medicine, University of Nis, 18000 Nis, Serbia; sanjavelickovicdr@gmail.com; 4 Department of Physiology, Faculty of Medicine, University of Nis, 18000 Nis, Serbia; milica.veljkovic@medfak.ni.ac.rs

**Keywords:** leukemia stem cell, oxidative phosphorylation, free fatty acid, transforming growth factor beta, lysophospholipids, autophagy

## Abstract

**Background/Objectives**: Quiescent leukemia stem cells (LSCs) are self-renewing, pluripotent cells that present a major obstacle to the successful curative treatment of chronic myeloid leukemia (CML). LSCs function independently of BCR::ABL1 signaling and persist following tyrosine kinase inhibitor treatment. The mechanisms enabling LSC survival are a central focus of current CML research. This review details the complex relationship between signaling pathways and discusses recent advancements in energy metabolism research within the pathogenesis of CML. **Discussion**: Energy metabolism is critical to the biology of CML LSCs. These cells depend on oxidative phosphorylation (OXPHOS) and mitochondrial homeostasis, utilizing fatty acid oxidation as their primary ATP source. Research highlights significant alterations in signaling networks, marked by a dynamic interplay among dominant pathways within the CML clone. While TGF-β-FOXO signaling maintains the self-renewal capacity of quiescent LSCs, proliferating mature CML cells rely heavily on glycolysis and the PI3K/Akt pathway. Furthermore, unique metabolic traits of LSCs underscore the impact of leukemic cell–microenvironment interactions in fostering a permissive niche. **Conclusions**: Fatty acid oxidation is critical to the survival and self-renewal of CML LSCs. This adaptation of mitochondrial function is closely linked to signaling alterations and entails an adjustment of mitochondrial respiration alongside stimulated OXPHOS. Emerging research unveils many potential targets within metabolic signaling that can be exploited to overcome these survival mechanisms, highlighting the disruption of mitochondrial energy support as a promising strategy to selectively eradicate CML LSCs.

## 1. Introduction

Quiescent leukemia stem cells (LSCs) form the foundation of residual disease in chronic myeloid leukemia (CML) due to their inherent insensitivity to tyrosine kinase inhibitors (TKIs). These self-renewing, pluripotent myeloid stem cells can re-initiate leukemogenesis following TKI discontinuation, representing a major obstacle to curative CML treatment [1,2,3]. While inhibition of BCR::ABL1 tyrosine kinase signaling effectively eliminates mature CML cells, it fails to eradicate persistent LSCs. Experiments with transgenic mouse models and patient-derived in vitro CD34(+) CML cells confirm that the survival of these stem cells is BCR::ABL1 kinase-independent [2,4,5].

The mechanisms enabling LSC survival during TKI treatment are a major focus of current research, alongside efforts to achieve treatment-free remission in these patients. To develop novel anticancer strategies that successfully target LSCs, it is crucial to understand CML pathogenesis and elucidate the mechanisms underlying LSC survival. These potentially curative approaches predominantly focus on BCR::ABL1-independent resistance mechanisms [1,2,6].

Research evidence suggests several BCR::ABL1-independent mechanisms of CML stem cell survival. These include the actions of cytokines and growth factors, the alternative activation of signaling pathways, enhanced lipid mediator signaling, and dysregulated immune responses. Additionally, alterations in the bone marrow (BM) microenvironment, epigenetic modifications (such as DNA methylation, histone acetylation, and miRNA expression), and alternative splicing have been implicated [1,2,4,7,8,9,10,11].

Alongside persistent LSCs, a subpopulation of progenitor stem-like CML cells can emerge during therapy, possessing acquired drug resistance (treatment-resistant cells). Their resistance primarily stems from genetic instability and secondary mutations within the BCR::ABL1 oncogene or other genes critical for cell-cycle regulation and maintenance [3]. A significant portion of this TKI resistance arises from mutations within the kinase domain of the BCR::ABL1 fusion protein, which can often be overcome by switching to alternative TKIs (e.g., nilotinib, dasatinib, and bosutinib) or other drugs that inhibit tyrosine kinases. Alternatively, resistance may develop from acquired mutations or the aberrant activation of pathways downstream of BCR::ABL1 kinase activity [4,7]. Another important issue to consider is the induction of antiproliferative effects in CML stem/progenitor cells by TKIs. By blocking BCR::ABL1 activity, other independent signaling pathways remain unopposed, leading to cell cycle arrest—a state known as induced quiescence (Table 1) [5,12].

Recent advances in understanding the unique aspects of LSC metabolism highlight the contribution of leukemic cell interactions, including induced modifications that shape a more permissive microenvironment for survival. A growing scientific field, known as immunometabolism, explores the interconnection between cell activation signaling and metabolic changes that further govern cell phenotype, function, and fate. Several cellular metabolites serve as cofactors, enhancers, or inhibitors of enzymes, transcription factors, and epigenetic modifiers involved in cell-cycle control and survival. Their activation network demonstrates that epigenetic and metabolic reprogramming are profoundly intertwined. Mitochondria are central to this process, given their involvement in energy production, redox reactions, cell death regulation, immune responses, and other physiological processes [1,2,3,4,8,9].

This comprehensive narrative review explores and discusses the complex relationship between signaling pathways and energy metabolism in CML, highlighting their profound effects on cellular behavior and survival. The review presents the current understanding of CML pathogenesis, focusing on recent and major findings that emphasize metabolic alterations and related intercellular interactions within the BM microenvironment. Furthermore, the article summarizes proposed novel biomarkers of treatment response alongside therapeutic strategies to address treatment resistance. To ensure a thorough synthesis, we utilized an integrative approach in selecting studies published in peer-reviewed journals, without limits on publication year or article type.

## 2. Discussion

### 2.1. Signaling Networks in the Pathogenesis of CML

The BCR::ABL1 fusion gene, generated by the chromosomal translocation t(9;22), is considered the cause of CML development. Detecting the BCR::ABL1 sequence is critical for CML diagnosis, while measuring transcript levels is used to monitor patient response, track treatment-free remission, and detect disease resistance or recurrence [6,7,8]. The BCR::ABL1 fusion protein exhibits enhanced dimerization and sustained tyrosine kinase activity, initiating a complex signaling cascade that dysregulates cellular homeostasis. This cascade accelerates the cell cycle, inhibits apoptosis, and impairs DNA repair, rendering cells largely independent of growth factors (Table 2) [4,7,28].

The initial phase of CML is characterized by the aberrant proliferation and survival of granulocytic cells, with neutrophils comprising a major portion of the CML clone. Later, the disease may progress into the blastic phase, marked by the accumulation of differentiation-arrested blast cells; this is driven by molecular alterations such as the loss or dysfunction of the essential transcription factor C/EBPα, including its post-transcriptional modification by the hnRNP E2 RNA-binding protein [4,7,28,29].

The BCR::ABL1 oncoprotein initiates a multitude of intracellular signaling pathways, either directly or indirectly. These include the RAS pathway, mitogen-activated protein kinases (MAPK), the MYC proto-oncogene, signal transducer and activator of transcription (STAT) transcription factors, and the phosphoinositide 3-kinase (PI3K)/Akt/mammalian target of rapamycin complexes (mTORC) [4,7,38]. The more recently described WNT/β-catenin/LEF-1 and Hedgehog (Hh) pathways are also important for the development of myeloid leukemias and LSC maintenance. The survival of the malignant clone is facilitated by the modulation of tumor-suppressor phosphatases and FOXO transcription factor signaling, among other mechanisms, as well as by the increased expression of anti-apoptotic proteins such as BCL-X(L). Furthermore, the expression of epigenetic modulators is often altered in this context [4,7,13,39].

BCR::ABL1 activity causes constitutive activation of downstream RAS signaling cascades, leading to MEK and MAPK activation, thereby supporting abnormal cell proliferation. The PI3K/Akt/mTORC1 signaling pathway is required for malignant transformation in CML and governs proliferation in the vast majority of mature CML cells. This pathway can be initiated by the BCR::ABL1 kinase or activated independently by other signaling mechanisms, such as STAT5, as demonstrated in imatinib-treated patients. Central to its downstream effects is the promotion of proliferation and cell survival via the activation of mTORC1 and the suppression of FOXO transcription factors [10,38].

**Table 2:** Major cytoplasmic and nuclear signaling events that regulate leukemia cell survival before and after TKI treatment.

Signaling Pathway	Before TKIs Treatment	After TKIs Treatment	References
The PI3K/AKT/mTOR signaling	Protein kinase B (Akt) is a central component of the PI3K pathway that controls downstream signaling networks, including mTORC1, FOXO, MYC, HIF-1α, and GSK3. Glycolytic enzyme hexokinase 2 (HK2) is a direct target of Akt; it regulates glycolytic intensity and promotes pro-survival cues. HK2 protects mitochondria from oxidative stress and promotes autophagy during glucose starvation. Glycolytic enzyme PKM2 inhibits apoptotic pathways and binds to STAT5A to induce cyclin D1 expression.	TKIs turn down the PI3K/AKT/mTOR pathway. HK2 disruption induces Ca2+ -dependent calpain activation while perturbing ROS balance and glutathione homeostasis, ultimately driving cell death. PKM2 knockdown—or its induced isoform switching to PKM1—triggers autophagic cell death via Bcl-2 disruption and elevated ROS levels.	[40,41,42,43,44,45]
The BCR::ABL1/STAT5 axis	STAT5 induces transcription of target genes (e.g., cyclin D, c-MYC, Bcl-2, and MMP-2). The STAT5B isoform serves as a primary driver of leukemogenesis, with the suppression of IFN-α/γ responses representing a key mechanism that facilitates malignant transformation. Maintaining high STAT5 levels promotes cell proliferation and survival.	TKIs normally shut down the STAT5 signaling.	[7,46,47]
The TGF-β/FOXO signaling	TGF-β/FOXO signaling is crucial for maintaining the self-renewal capacity of LSCs. This network acts in conjunction with Smad2/3 proteins. TGF-β1 modulates the tumor microenvironment and immune system—particularly Tregs. BCR::ABL1 increases TGF-β/Smad-dependent transcriptional activity; additionally, extrinsic (stromal) TGF-β contributes to CML pathogenesis.	Upon TKI treatment, active FOXO upregulates BCL6 in CD34+ CML cells, which represses key targets—including Cyclin D2, Bcl-xL, Arf, and p53—thereby protecting LSCs from TKI toxicity. In contrast, BCL6 remains repressed in untreated CML via BCR::ABL1-dependent signaling.	[5,12,18,19,20,21,40,48,49,50,51,52,53]
The LKB1–AMPK pathway	AMPK regulates metabolic pathways by inhibiting mTOR, suppressing ATP-consuming processes, and inducing AMPK-dependent cytoprotective autophagy and mitochondrial biogenesis. The cascade promotes mitochondrial respiration (OXPHOS) and lipid utilization (FAO).	TKI treatment triggered AMPK-mediated autophagy in proliferating CML cells, forcing them to rely on mitochondrial respiration for survival. Autophagy protects LSCs following TKI and may contribute to disease persistence.	[54,55,56,57,58,59,60,61,62,63]
The Wnt/β-catenin signaling	It is required for the self-renewal of both normal and neoplastic stem cells. It supports transcription of target genes that drive the expansion of leukemic progenitors.	The WNT/β-catenin pathway is significantly involved in TKI resistance. TKI-induced downregulation of miR-29 increases CD70 expression, thereby enhancing downstream Wnt signaling pathway activation.	[3,13,21,24,28,29,30,31,32,61]
The Hedgehog (Hh, SHH/SMO) signaling	The Hh signaling controls vital downstream cell-cycle and survival regulators (e.g., Cyclin D1 and Bcl2).	The Hh signaling cascade is highly active in LSCs, where it mediates survival independently of BCR-ABL1 kinase activity.	[33,34]
The protein kinase C (PKC) pathway	Crosstalk between PKC and BCR::ABL1 signaling promotes leukemogenesis. Receptor-stimulated activation of phospholipase C drives PKC activity, which subsequently activates CRAF to engage the downstream RAF/MEK/ERK cascade.	LSCs exhibit high PKCη transcript and protein levels. PKCη activates the RAF/MEK/ERK signaling, promotes cell proliferation, and inhibits apoptosis. PKC-β overexpression participates in the resistance. ERK1/2 pathway mediates Alox5 upregulation by PKC-β and induces TKI-insensitivity through inactivation of PTEN.	[23,32,49]
The SIRT1/PGC-1α axis	SIRT1 overexpression in LSCs is regarded as a key survival mechanism activated downstream of BCR::ABL1/STAT5 signaling. However, SIRT1 activation is not entirely dependent on BCR::ABL1 kinase activity. SIRT1/PGC-1α axis drives metabolic alterations—a shift from glycolysis toward OXPHOS. SIRT1 overexpression correlates with enhanced conversion of LC3, a key ubiquitin-like protein.	SIRT1 overexpression is involved in the mitochondrial metabolic rewiring associated with LSC maintenance and TKI resistance. SIRT1/PGC-1α axis enhances OXPHOS capacity and TKI resistance in LSCs.	[26,64,65,66,67]

GSK3—glycogen synthase kinase 3, HK2—hexokinase 2, LKB1—liver kinase B1, PKM2—pyruvate kinase muscle type 2, Alox5—Arachidonate 5-lipoxygenase, PTEN –phosphatase and tensin homolog, a tumor suppressor, SIRT—sirtuin.

**Figure 1:**
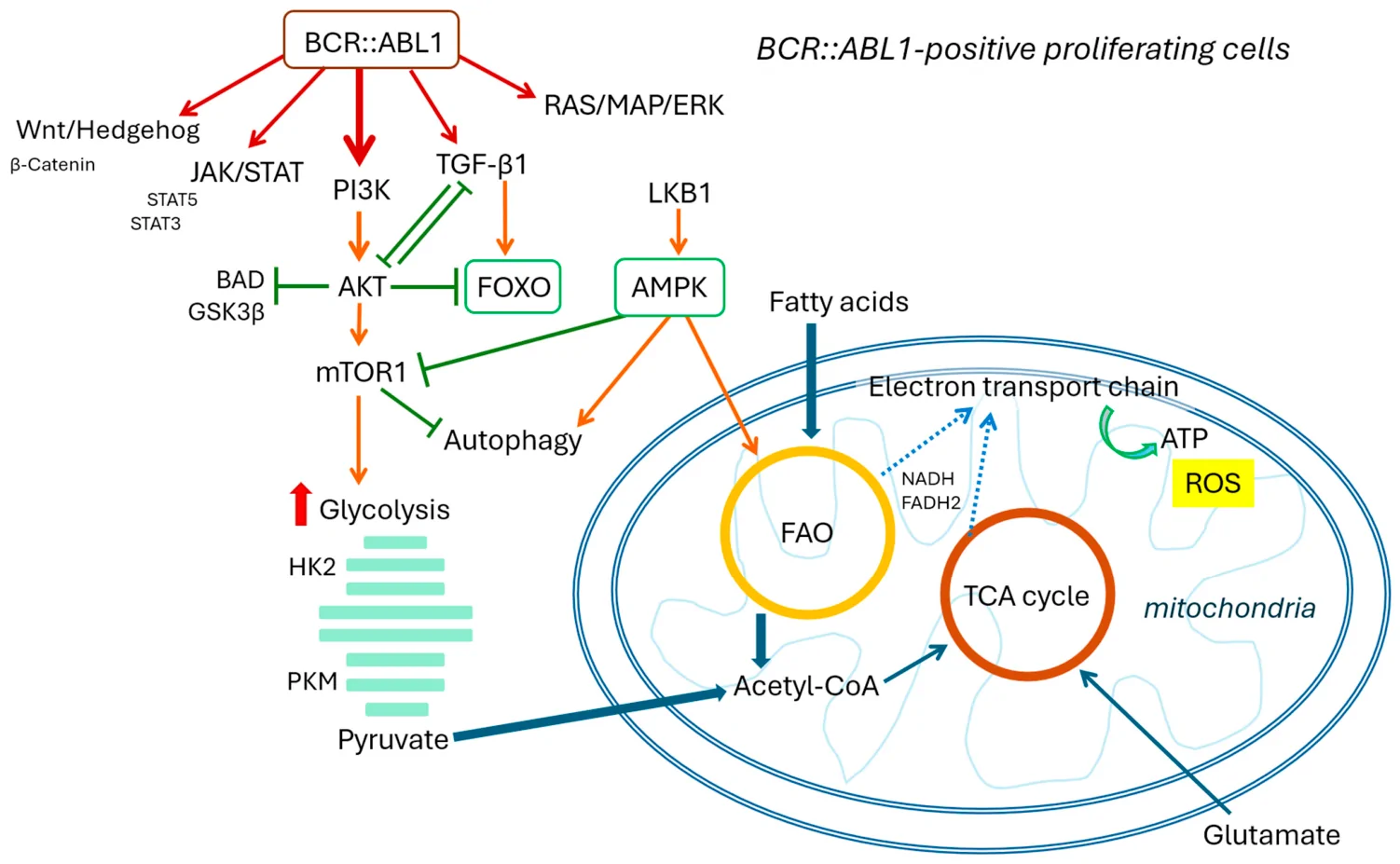
Schematic overview of signaling networks in BCR::ABL1-positive proliferating CML cells. The BCR::ABL1 oncoprotein initiates several signaling pathways (depicted by red arrows). Proliferating mature CML cells rely heavily on the PI3K/Akt pathway for glycolysis and survival, a pathway typically associated with the inactivation of FOXO transcription factors. Autophagy can be upregulated by AMPK, elevated ROS levels, or other stimuli, whereas the PI3K/mTOR pathway generally suppresses its activation. AMPK signaling functions as a central cellular energy sensor, modulating metabolic pathways by inhibiting mTOR, suppressing ATP-consuming processes, and enhancing fatty acid oxidation (FAO). Red arrows show direct stimulation by the oncogene. Orange arrows follow intracellular stimulating actions, while green T-headed arrows reflect a blocking effect. Blue arrows depict stimulating signaling in metabolic pathways and mitochondria. Light blue lines below glycolysis represent glycolytic enzymes; HK2 and PKM denote hexokinase 2 and pyruvate kinase muscle isoform, respectively. Bright red arrow depicts enhanced glycolysis.

Phosphoinositide 3-kinase, a lipid kinase, mediates activation of phosphoinositide-dependent protein kinase 1 (PDK1), which together with mTORC2 activates serine/threonine kinase Akt (protein kinase B)—a central kinase in the pathway that controls downstream signaling networks, including mTORC1, FOXO, MYC, HIF-1α, and glycogen synthase kinase 3 (GSK3). Thus, the PI3K pathway plays key roles in regulating proliferation, survival, metabolism, angiogenesis, and autophagy, while suppressing apoptosis (e.g., via inactivation of the proapoptotic molecule BAD). Furthermore, PI3K/Akt signaling significantly affects glucose metabolism and mitochondrial oxidative phosphorylation (OXPHOS), as discussed in the following sections (Figure 1) [38,39,40,68].

The transcription factors STAT1, STAT3, and STAT5 activate key downstream pathways in CML. The BCR::ABL1 oncoprotein directly activates STAT5, which translocates to the nucleus to induce transcription of target genes (e.g., cyclin D, c-MYC, Bcl-2, and MMP-2). Due to the constitutive activity of the oncoprotein, STAT5 remains continuously activated. Maintaining high STAT5 levels promotes cell proliferation and survival [7,46]. The STAT5B isoform serves as the primary initiator of leukemogenesis, with the suppression of IFN-α/γ responses representing one of its mechanism that enables malignant transformation [47]. Furthermore, high STAT5 expression can lead to imatinib resistance and disease progression [4,69].

Although STAT3 and STAT5 are activated by distinct mechanisms and bind to different genomic loci, they can exhibit significant functional crosstalk. Beyond directly binding to gene promoter regions, their actions involve combined interactions with other transcription factors, epigenetic regulation, and shaping of the chromatin landscape [46,47,69,70,71,72]. Additionally, unphosphorylated STAT proteins can mediate non-canonical processes, including transcriptional modifications (differing from those of phospho-STATs), alterations in mitochondrial function, and changes in chromatin structure [46,73]. Moreover, STAT3 activation can be driven by external stimuli independently of BCR::ABL1, which may contribute to TKI resistance [4,22].

Cross-talk among the PI3K/Akt, MAPK, TGFβ, and WNT signaling pathways regulates the switch between self-renewal and differentiation in pluripotent stem cells. This network acts in conjunction with Smad2/3 proteins—the primary signal transducers of TGFβ superfamily receptors. When PI3K/Akt is inhibited and the WNT pathway is activated, elevated Smad2/3 activity collaborates with β-catenin to target genes required for early differentiation [4,7,38,48].

#### 2.1.1. Signaling Pathways in BCR::ABL1-Independent Resistance in CML

Chronic myeloid leukemia LSCs differ biologically from normal HSCs through their heightened proliferative capacity, altered oxidative metabolism, and resistance to apoptosis. Leukemic stem cells survive primarily through kinase-independent pathways. Studies have identified aberrant expression of cell surface molecules on CML LSCs alongside high heterogeneity among quiescent LSCs across genetic, epigenetic, and transcriptional mechanisms. Accordingly, a number of alternative, BCR::ABL1-independent survival pathways are being investigated in CML stem and progenitor cells, including epigenetic alterations and BM microenvironment signals (Table 2) [11,23,49].

The protein kinase C (PKC) pathway plays an important role in leukemogenesis, cooperating with BCR::ABL1 to promote cell proliferation and survival. Investigating BCR::ABL1-independent imatinib resistance, Ma et al. identified elevated expression of PKCη, a PKC family member that drives downstream activation of the RAF/MEK/ERK axis. Mechanistically, PKCη activates CRAF, sustaining RAF/MEK/ERK signaling to enhance cell survival and block apoptosis. Importantly, CML stem cells exhibit high PKCη transcript and protein levels, which likely contribute to their intrinsic imatinib resistance. Overexpression of another PKC family member, PKC-β, was also found to be significantly related to TKI insensitivity in CML cell lines. This mechanism involves the ERK1/2 and arachidonate 5-lipoxygenase (ALOX5) signaling pathways, which mediate the inactivation of PTEN, a critical tumor suppressor gene [23,30,49].

Signaling pathways that bypass the BCR::ABL1 kinase often rely on WNT/β-catenin and Hedgehog signaling in LSCs. Deregulation of the WNT/β-catenin pathway is implicated in the pathogenesis of various neoplasia, including leukemogenesis. Upon binding to its receptor, WNT inhibits β-catenin phosphorylation, thereby preventing its degradation and allowing the transcription of target genes that drive the expansion of leukemic progenitors. CML LSCs exhibit aberrant control of β-catenin signaling, which is further supported by the BCR/PI3K/AKT pathway. A gene expression profiling study found that deregulation of the WNT pathway is largely responsible for TKI resistance and carries a negative prognostic value. However, the loss of β-catenin impairs the self-renewal of both normal and CML cells. Additionally, the bone morphogenetic protein (BMP) signaling pathway, typically originating from the BM, can activate pro-survival mechanisms in BCR::ABL1 oncogene-independent fashion. This pathway is highly deregulated in CML, with BMP4 directly or indirectly stimulating the WNT pathway [3,23,28,31,32].

The Hedgehog (Hh, SHH/SMO) signaling cascade is highly active in LSCs, where it mediates survival independently of BCR-ABL1 kinase activity. Hedgehog ligands secreted by BM stromal cells ultimately upregulate vital downstream cell-cycle and survival regulators (e.g., Cyclin D1 and Bcl2). Hedgehog signaling is activated in LSCs through the upregulation of the transmembrane receptor Smoothened (SMO). Higher SMO expression has been detected in all Ph+ cells in the BM compared to normal hematopoietic cells in mice. However, ABL inhibition in murine BCR-ABL-positive BM cultures decreases Smo transcript levels by only 20%, indicating that additional, oncogene-independent mechanisms account for its elevated expression. It is hypothesized that the oncogene may induce constitutive Hh signaling or cause hyperresponsiveness to Hh ligand stimulation in these cells. Additionally, SMO inhibition has been shown to reduce LSCs in vivo [33,34].

Epigenetic dysregulation is a well-established and common abnormality in tumorigenesis. Epigenetic changes are also implicated in CML progression and TKI resistance. Among others, expression of the SIRT1 deacetylase is higher in human CML CD34+ cells than in normal cells, a feature that is partially independent of BCR::ABL1 activity. By regulating the acetylation of several transcription factors, SIRT1 can significantly affect cellular survival (see Section 2.2.6.) [34,74].

The mitochondrial translation pathway represents a molecular alternative to the BCR::ABL pathway that can be exploited for LSC eradication [64]. A transcriptomic analysis of the three-node miRNA feed-forward loop in CML revealed a novel mechanism governing mitochondrial metabolic rewiring and cell senescence in CML leukemogenesis with novel SIRT1 connections. Specifically, a SIRT1-dependent mechanism regulating mitochondrial metabolism was found to involve a gene regulatory network composed of miR-transcription factors—precisely miR-34a, miR-205, ETS1, and/or GFM1 mRNA. Disrupting mitochondrial translation interferes with the cellular ability to perform terminal oxidation [35,65,75].

Soluble factors and cellular components from the BM stroma can induce adaptive signaling in adjacent leukemia cells, elevating apoptotic thresholds and sustaining survival. Several cytokines are being investigated as potential stimulators of LSC survival, suggesting that an inflammatory microenvironment contributes to leukemogenesis. Whole-transcriptome single-cell analysis of CML LSCs identified pathways of potential therapeutic interest, including the transforming growth factor-beta (TGF-β) and tumor necrosis factor-alpha (TNF-α) pathways, which were characterized by a marked overexpression of inflammation-associated genes (see Section 2.1.2 and Section 2.3.) [4,23,76,77,78].

#### 2.1.2. Cytokines in CML Pathogenesis

Increased levels of circulating proinflammatory cytokines have been reported in patients with CML. Cytokines and chemokines are secreted both by leukemic cells and by non-malignant immune and stromal cells within BM niches [14,76,77,78,79]. Research evidence suggests that locally produced cytokines favor malignant hematopoiesis by activating distinct signaling pathways—such as PI3K/Akt/mTOR and STAT3—thereby supporting disease progression and TKI resistance. Consequently, cytokine-directed interventions address BCR::ABL1-independent mechanisms and represent a promising strategy for future CML management (Table 3) [4,25,76,77,78,80].

Tumor necrosis factor alpha (TNF-α) has been extensively investigated and shown to play a significant role in CML pathogenesis [14,76,77,78,79]. For instance, Giustacchini et al. demonstrated that, compared to normal hematopoietic cells, LSCs express higher levels of inflammation-related genes, such as TNF-α and TGF-β. Single-cell transcriptomics further revealed that these two cytokines are associated with increased stem cell quiescence and TKI resistance [79].

CML stem and progenitor cells produce higher levels of TNF-α than their normal counterparts. This provides them with survival signals through the NF-κB/TNF-α feedback loop, IL-3 expression, and GM-CSF β-chain receptor signaling. Importantly, TNF-α production is independent of BCR::ABL1 kinase activity. Additionally, baseline TNF-α levels at diagnosis were significantly higher in CML patients who failed to achieve remission after 6 months of imatinib therapy compared to those who responded successfully [4,76,77,78,79,81]. TNF-α has several important metabolic implications. Specifically, it can stimulate lipolysis, regulate lipid droplet dynamics, and promote triglyceride accumulation in differentiated human adipocytes, which comprise a substantial portion of adult BM. Lipolysis is enhanced through the activation of MEK/ERK and cAMP-dependent protein kinase A. Through these same kinases, TNF-α suppresses peroxisome proliferator-activated receptor gamma (PPAR-γ) transcriptional activity and downregulates the expression of cell death-inducing DFF45-like effector C (CIDEC), an important negative regulator of lipolysis [82,83,84].

A recent work identified JAK1-STAT3-activating signaling and IL-6 as responsible for TKI-resistant CML cases, proposing novel, potentially curative therapeutic targets in CML. Indeed, the combined use of BCR::ABL1 and JAK1/2 selective inhibitors reduced CML cell proliferation and induced apoptosis, even in quiescent LSCs [25]. Another study identified MCP-1 and IL-6 as novel, strong, and predictive plasma biomarkers for treatment-free remission in CML, among 20 cytokines tested. MCP-1 and IL-6 levels were markedly increased in CML patients in treatment-free remission, whereas low MCP-1/IL-6 levels were associated with poorer relapse-free survival [85].

Transforming growth factor beta (TGF-β) is a pleiotropic cytokine that regulates a wide array of cellular functions, and its aberrant signaling is implicated in numerous diseases. Plasma TGF-β levels are elevated in patients with CML; notably, treatment with the TKI dasatinib significantly reduces these levels within 3 months. However, higher baseline TGF-β levels correlate with a lower likelihood of achieving a favorable therapeutic response. This pattern may be driven by the relationship between TGF-β1 and regulatory T cells (Tregs). Specifically, dasatinib-mediated inhibition of Treg suppressive function is accompanied by decreases in both Treg frequency and serum TGF-β1 levels. Consequently, TGF-β levels have been proposed as a predictive biomarker in CML [48,86,87].

**Table 3:** Cytokines implicated in the pathogenesis of CML.

Cytokine	Cellular Source	Biological Function in CML	References
Tumor necrosis factor-alpha (TNF-α)	Primarily produced by activated macrophages and monocytes. Secreted by T cells, B cells, dendritic cells, neutrophils, mast cells, adipocytes, BMSCs. CML stem/progenitor cells.	Supports the survival of malignant cells via the NF-κB/p65 pathway, the GM-CSF common β-chain receptor, and IL-3 expression. Enhances LSC growth via CXCR2 signaling. Stimulates lipolysis & regulates lipid droplet dynamics, in differentiated human adipocytes.	[8,9,15,76,77,78,79,88,89,90]
Interleukin 6 (IL-6)	Macrophages, BMSCs, T cells, osteoblasts, adipocytes. CML stem/progenitor cells.	Establishes a paracrine feedback loop with BCR::ABL1 that sustains leukemia development. Interacts with the JAK/STAT signaling pathway. Increased IL-6 levels drive LSC expansion.	[9,15,25,76,77,78,79,85,88,89,90]
Interleukin 1 beta (IL-1β)	Macrophages, monocytes, dendritic cells, B cells, neutrophils. CML stem/progenitor cells.	IL-1 receptors are elevated on the surface of LSCs, underscoring the influence of inflammatory signals. Elevated IL-1β helps CML cells evade apoptosis.	[8,9,15,23,30]
Interleukin 3 (IL-3)	T cells, mast cells, and BMSCs. CML stem/progenitor cells.	JAK2/STAT5 activation and growth factor (GF)-independent growth	[76,77,78,79]
Increased 8 (IL-8)	Myeloid precursors, BMSCs, MSCs, resident macrophages, dendritic cells.	IL-8 exhibits both autocrine and paracrine effects, thereby facilitating CML LSCs’ adhesion to MSCs and sustaining their survival.	[66,91]
Transforming growth factor beta (TGF-β)	Primarily produced by MSCs and osteoblasts. CML stem/progenitor cells. Also, stored in the bone matrix.	Regulates phosphorylation of Akt, facilitates activity of FOXO3a transcription factor, thereby potentiating LSC maintenance. TGF-β as an extrinsic (stromal) source contributes to CML pathogenesis. An autocrine loop was shown for CML-derived exosome-associated TGF-β1, which was followed by activation of ERK/Akt/NF-κB signaling and anti-apoptotic pathways. TGF-β releases MMP-9 and alters the BM niche.	[11,13,48,50,51,55,78,86,87,91,92,93]
Monocyte chemoattractant protein-1 (MCP-1/CCL2)	Bone marrow stromal cells, MSCs, osteoblasts, endothelial cells, adipocytes. Mast cells of malignant clone.	Recruits monocytes and macrophages to the BM and spleen. Creates a chronic inflammatory state that supports tumor progression and angiogenesis.	[25,76,77,78,85,88,89,90]

Bone marrow stromal cells (BMSCs), mesenchymal stem cells (MSCs), leukemia stem cell (LSC), granulocyte/macrophage-colony stimulating factor (GM-CSF), matrix metalloproteinase-9 (MMP-9).

#### 2.1.3. Comparison of Major Signaling Pathways in CML LSCs vs. Mature CML Cells

In fast-proliferating CML cells, BCR::ABL1-mediated activation of the PI3K/Akt/mTORC1 pathway drives cellular metabolism and survival. In these cells, BCR::ABL1 activity is associated with the post-translational inactivation of FOXO transcription factors (FOXO1, FOXO3a, and FOXO4). Conversely, in quiescent CML LSCs, Akt phosphorylation levels decrease, whereas TGF-β signaling and its downstream target FOXO remain active. Furthermore, FOXO phosphorylation levels are higher in CML cells than in normal CD34+ cells. Together, these findings underline TGF-β/FOXO signaling as crucial for maintaining the self-renewal capacity of LSCs [5,12,40,50,51].

The PI3K and TGF-β signaling networks appear to diverge in governing LSC fate. Nevertheless, the complex interplay among TGF-β, Akt, and FOXO activity in both normal hematopoietic stem cells (HSCs) and LSCs remains highly context-dependent. Through cell-intrinsic and cell-extrinsic mechanisms, TGF-β1 modulates the tumor microenvironment and immune system—particularly Tregs—thereby exerting dual pro- and anti-tumor effects [5,11,12,50,51,87].

In experiments with leukemia-initiating cells (LICs), TGF-β was associated with decreased Akt phosphorylation and increased nuclear localization of FOXO3a, the predominant FOXO isoform in these cells. On the other hand, FOXO3a is strongly regulated by PI3K/Akt signaling: active Akt phosphorylates nuclear FOXO3a, causing its export to the cytoplasm and inhibiting its transcriptional activity, where it is subsequently degraded. In quiescent CML LSCs, Akt activity is low; as a result, FOXO3a remains in the nucleus, where it can induce G1 cell cycle arrest or apoptosis [12,50].

Experiments using mouse models demonstrated that Foxo3a deficiency impedes the propagation of CML cells. Specifically, the pool of Foxo3a -/- LICs significantly decreased following serial BM transplantation. Similar reductions in colony-forming capacity and confirmation of the critical role of TGF-β/FOXO signaling were observed in primary human CML LICs [50]. Functionally, FOXO3a undergoes various post-translational modifications that fine-tune its activity, stability, DNA binding, and intracellular localization [51,94,95,96]. An interconnection between the PI3K pathway and the cellular localization of the FOXO1 transcription factor has also been described in CML. TKI-resistant cells with activated PI3K/Akt signaling exhibit increased Akt-mediated FOXO1 phosphorylation, leading to its cytoplasmic sequestration and reduced nuclear accumulation. Upon PI3K/Akt pathway inhibition, FOXO1 translocates to the nucleus [4,97].

Research also indicates that FOXO transcription factors underpin the antiproliferative activity of TKIs. By suppressing PI3K/Akt signaling, TKIs induce unopposed FOXO activation, triggering cell cycle arrest. While this suppresses proliferation, it also induces a protective state in CML cells against genetic instability and apoptosis. Pellicano et al. demonstrated that dasatinib significantly reduces FOXO phosphorylation and promotes the nuclear translocation of total FOXO1 and FOXO3a, thereby enhancing their transcriptional activity. Furthermore, shRNA-mediated knockdown of FOXO3a sensitized CML cells to TKI-induced apoptosis [5].

Key FOXO target genes include CCND1 (Cyclin D1), ATM, CDKN1C (p57), and BCL6. As a central mediator of FOXO signaling, BCL6 supports CML stem cell survival under TKI pressure. Upon TKI treatment (24 h), active FOXO upregulates BCL6 in CD34+ CML cells. In turn, BCL6 represses key targets—including Cyclin D2, Bcl-xL, Arf, and p53—thereby protecting LSCs from TKI toxicity. In contrast, BCL6 remains repressed in untreated CML via BCR::ABL1-dependent signaling [5,52,53].

Notably, TGF-β exerts distinct effects on FOXO3a activation and localization in LICs versus non-LICs (progenitor cells), differential pathways that guide the survival of these two leukemic sub-populations. In non-LICs, FOXO3a activation following TKI treatment leads to cell cycle arrest and apoptosis via TNF-α- or p53-dependent pathways [5,50,98].

In a genome-wide transcriptomic comparison, the authors observed greater similarity in expression patterns between CML LSCs and normal HSCs than between CML LSCs and CML progenitor cells. Nevertheless, the study reports a downregulation of pro-differentiation and TGF-β/BMP signaling pathways in CML LSCs (CD34+ CD38− ALDHhigh [aldehyde dehydrogenase activity]) relative to normal HSCs. This was accompanied by a marked suppression of TGF-β/BMP transcriptional output alongside the activation of pathway antagonists [11].

Moreover, co-culture systems with stromal cells modulate the effects of TGF-β and other soluble factors, underscoring the importance of microenvironmental interactions in tuning malignant cell behavior. Actually, the colony-forming capacity of LSCs was unaffected when cells were cultured in a stroma-free system with TGF-β inhibitors, suggesting that an additional extrinsic (likely stromal) source or mechanism of TGF-β contributes to CML pathogenesis [11,50,51].

#### 2.1.4. The BCR::ABL1 Oncoprotein Degradation

Recent research underlines the BCR::ABL1 degradation process as a potent therapeutic strategy—one superior to merely inhibiting fusion protein activity and promising for overcoming TKI resistance. Several key regulatory molecules mediate the ubiquitin-proteasome degradation pathway for both mature and immature BCR::ABL1 proteins. Among these, heat-shock proteins (HSPs) play a central role in modulating ubiquitination and subsequent proteasomal degradation. Additionally, the BCR::ABL1 fusion protein can be degraded via the autophagy-lysosome pathway and caspase-mediated cleavage. Mechanistically, alterations in chaperone and co-chaperone dynamics direct BCR::ABL1 to the proteasome, destabilizing or dephosphorylating the protein and rendering it more susceptible to degradation. Several compounds have demonstrated efficacy in promoting BCR::ABL1 degradation by compromising its structural stability. Furthermore, multiple HSP90 inhibitors effectively disrupt the interaction between BCR::ABL1 and HSP90, accelerating its clearance. Moreover, Yang and colleagues demonstrated that heat stress facilitates BCR::ABL1 oligomerization and triggers its degradation via c-Cbl-mediated, non-canonical K27-linked ubiquitination involving the PI3K-p85α pathway in Ph+ ALL patients [99,100,101,102,103]. However, this strategy may be less effective against quiescent LSCs, as their survival is independent of BCR::ABL1 kinase activity and they remain refractory to TKIs. Nevertheless, various autophagy-related mechanisms are being actively investigated as potential therapeutic targets in LSCs [1,2,3,4,5,104], which will be discussed in detail below.

### 2.2. Energy Metabolism in CML Cells

Recent studies report that cancer stem cells exhibit metabolic flexibility, enabling them to alternate between glycolysis and OXPHOS in response to environmental cues to secure energy, evade apoptosis, and escape immune surveillance. This metabolic switch is intrinsic to leukocytes and occurs physiologically during activation, allowing them to adapt their energy demands to the inflammatory response [3,12,40,54,105].

Proliferating BCR::ABL1-positive cells show high glycolytic activity. However, imatinib administration markedly suppresses cytosolic glycolysis, accompanied by a shift toward increased mitochondrial fatty acid oxidation (FAO) and elevated tricarboxylic acid (TCA) cycle intermediates. This mitochondrial energy supply appears essential for sustaining BCR::ABL1-active CML cell survival during TKI exposure [41,106,107].

#### 2.2.1. Metabolic Roles of the PI3K/Akt/mTOR Axis

As previously noted, the majority of proliferating mature CML cells exhibit robust BCR::ABL1 signaling accompanied by activation of the PI3K/Akt/mTORC1 pathway, which exerts a profound impact on malignant cell metabolism. Broadly, PI3K/Akt signaling promotes glycolysis by upregulating glucose transporters (e.g., the high-affinity glucose transporter GLUT1) and key glycolytic enzymes, while suppressing glycogen synthase kinase 3 (GSK3). PI3K/Akt signaling promotes de novo lipid synthesis and directly stimulates cholesterol and fatty acid (FA) biosynthesis, while inhibiting gluconeogenesis and FAO, in part by directly phosphorylating peroxisome proliferator-activated receptor gamma coactivator 1-alpha (PGC-1α). Additionally, it modulates the pentose phosphate pathway (PPP), mitochondrial oxidative phosphorylation (via complex I activity), and redox homeostasis by replenishing the cytosolic pool of NADPH, a key reducing agent. Overall, the PI3K/Akt pathway acts to store energy and mitigate oxidative stress [10,40,68,108,109,110].

Protein kinase B (Akt) is a central component of the PI3K pathway, playing a key role in regulating cellular metabolism through its effects on enzymes, nutrient transporters, and signaling networks. Akt substantially influences enzymes that regulate glycolysis. For instance, the glycolytic enzyme hexokinase 2 (HK2) is a direct target of Akt; this interaction exerts bidirectional effects on glycolytic intensity and promotes pro-survival cues [40,42,111]. Specifically, HK2 binds to the voltage-dependent anion channel on the outer mitochondrial membrane (OMM), inhibiting apoptosis by limiting the opening of the mitochondrial permeability transition pore (mPTP) [40,111,112]. HK2 also binds to mitochondria-associated membranes (MAMs)—the communication sites between the OMM and ER—where it helps regulate Ca2+ turnover. The displacement of HK2 from MAMs promotes Ca2+ release, triggering Ca2+-dependent calpain activation and ultimately cell death [40,43]. Under hypoxic conditions, another example of the Akt/HK2 axis involves HK2 forming a complex with fructose-2,6-bisphosphatase (TIGAR, a p53-inducible protein). This complex amplifies the PPP, thereby boosting NADPH production and suppressing mitochondrial ROS generation [40,113]. While HK2 protects mitochondria from oxidative stress, it also promotes autophagy during glucose starvation [42,44].

In CML, the detachment of HK2 from mitochondria promotes collateral sensitivity in multidrug-resistant cells that rely primarily on glycolysis for ATP production. This detachment disrupts ROS levels and glutathione homeostasis, ultimately increasing intracellular drug accumulation and inducing cell death. Consequently, manipulating HK2 represents a promising therapeutic strategy to overcome resistance in chemotherapy-refractory CML patients [114].

Pyruvate kinase (PK) is another key regulatory glycolytic enzyme modulated by the PI3K pathway. Pyruvate kinase exists in four tissue-specific isoforms, with the PKM2 (muscle type 2) isoform expressed in all proliferating cells, including tumors [40,41,115]. The PI3K pathway regulates PKM2 via the mTOR/c-Myc/hnRNP/PKM signaling cascade. Additionally, under IGF-1-induced PI3K activation, Akt can directly interact with and phosphorylate PKM2 [40,41,115]. In its dimeric or “low-activity” form, PKM2 acts as a protein kinase, whereas the tetrameric form functions as an active pyruvate kinase [116]. Consequently, dimeric PKM2 diverts glycolytic intermediates toward anabolic pathways for the biosynthesis of amino acids, lipids, and nucleotides [117,118]. Furthermore, dimeric PKM2 can translocate to the nucleus, mitochondria, or extracellular space. Upon nuclear translocation, PKM2 binds to STAT5A to induce cyclin D1 expression [40,107,118,119].

Studies have reported multiple binding partners of PKM2 and their associated effects on gene transcription, epigenetic and post-translational modifications, and exosome-mediated communication. Consequently, PKM2 is recognized as a key glycolytic enzyme involved in intercellular communication with significant potential for microenvironment reprogramming [117,118]. High expression of PKM2 in cancer cells plays a specific role in their metabolic reprogramming toward aerobic glycolysis [40,107,118,119]. In CML, PKM2 levels are significantly elevated, promoting glycolysis, biosynthetic pathways, redox homeostasis, cell survival, and drug resistance. Indeed, higher PKM2 expression is observed in TKI-resistant primary CML cells, whereas PKM2 knockdown reduces glycolysis and lactate production, suppresses cell growth, and induces apoptosis [120,121].

As a phosphotyrosine-binding protein, PKM2 interacts with several key partners, including the BCR::ABL1 kinase, with evidence suggesting reciprocal modulation between them. By interacting with Bcl-2 under oxidative stress, or with p53 in the presence of the MDM2 oncogene, PKM2 inhibits apoptotic pathways. Conversely, PKM2 knockdown—or its induced isoform switching to PKM1—triggers autophagic cell death via Bcl-2 disruption and elevated ROS levels [41,45]. Additionally, PKM2 phosphorylates STAT3, enhancing its transcriptional activity toward target genes such as MEK5 [116,117,118,122]. Interestingly, extracellularly secreted dimeric PKM2 promotes colon cancer cell migration by activating the PI3K/Akt and Wnt/β-catenin signaling cascades [123].

#### 2.2.2. CML Stem Cell Metabolism

Unlike normal HSCs and rapidly proliferating CML cells, CML LSC metabolism relies on increased OXPHOS and FA metabolism. A genome-wide comparative study demonstrated altered cellular functions in CML LSCs relative to normal HSCs, highlighting the upregulation of gene sets related to oxidative metabolism—including the mitochondrial inner membrane, electron transport chain (ETC), mitochondrial matrix, NADH-to-ubiquinone activity, and the Krebs/TCA cycle [11]. Nevertheless, LSCs remain metabolically less active than the bulk of their clonal progeny. Compared to healthy HSCs, LSCs exhibit metabolic flexibility by utilizing diverse fuel sources, including glucose, fatty acids, amino acids, and anaplerotic substrates such as glutamate [3,124]. Evidence also indicates that distinct CML LSC subpopulations possess unique metabolic profiles [15].

Using single-cell RNA sequencing and metabolomic profiling, recent work identified a subset of deeply quiescent LSCs. To avoid oxidative stress and maintain low metabolic activity, these persistent LSCs suppress mitochondrial complex I while relying on FAO for energy, allowing them to persist during therapy. Furthermore, the study highlights the potential of a CBP/β-catenin antagonist to disrupt this quiescence and drive differentiation, thereby restoring sensitivity to TKIs [125].

Chronic phase CML LSCs highly express mitochondrial respiratory chain genes and exhibit upregulated mitochondrial respiration and increased TCA cycle flux. Additionally, several studies show high ROS levels in these cells, which may be related to increased mitochondrial respiration [3,55,75,126,127]. Moreover, new evidence points to significant roles for lipid mediators, such as lysophospholipid (LysoPL) and branched-chain amino acid (BCAA) metabolism, in LSC survival, stemness, and TKI resistance [10,85].

Fatty acid and amino acid metabolism significantly contribute to maintaining OXPHOS in LSCs, with FAO playing a crucial role in their survival and self-renewal. FAO yields key metabolic intermediates that drive energy production while coordinating intracellular signaling, gene transcription, and protein function. Primarily, elevated FAO supports OXPHOS by supplying acetyl-CoA to the TCA cycle, thereby enhancing ATP production [15,54,56,73,127].

Furthermore, acetyl-CoA can exit mitochondria through the citrate-pyruvate cycle. Under glucose-deprived conditions, FAO-derived acetyl-CoA is utilized to produce cytosolic NADPH, among others, thereby compensating for the reduced PPP-derived NADPH. Acetyl-CoA is used to form citrate and malate, which are then converted by isocitrate and malate dehydrogenases, respectively, to produce NADPH. This way, NADPH significantly supports antioxidant regeneration, ameliorates redox imbalance, and helps in the repression of ROS-induced apoptosis [54,56,57,58,59,60].

Furthermore, acetyl-CoA can exit the mitochondria via the citrate-pyruvate shuttle. Under glucose-deprived conditions, FAO-derived acetyl-CoA is utilized to generate cytosolic NADPH, thereby compensating for the decline in PPP-derived NADPH. Specifically, acetyl-CoA contributes to the formation of citrate and malate, which are subsequently converted by cytosolic isocitrate dehydrogenase and malate dehydrogenase to produce NADPH. The resulting NADPH supports antioxidant regeneration, mitigates redox imbalance, and suppresses ROS-induced apoptosis [54,56,57,58,59,60]. Moreover, increased production of acetyl-CoA enhances histone and protein acetylation by providing the acetyl donor required by histone acetyltransferases. This modification promotes chromatin relaxation, thereby increasing DNA accessibility and modulating gene expression [56].

Increased FAO in CD34+ CML cells is associated with elevated acylcarnitine levels and the accumulation of metabolites that enrich both the TCA cycle and the amino acid pool. Additionally, TCA cycle flux is supported by enhanced activity of the anaplerotic enzyme pyruvate carboxylase, which catalyzes the conversion of pyruvate to oxaloacetate [128]. Comparative metabolic profiling reveals that CD34+ stem-cell-enriched CML cells exhibit elevated levels of glycerol-3-phosphate and carnitine, alongside depleted FFAs such as oleic and stearic acids, relative to differentiated CD34- CML cells [75].

Research shows that FAO plays a critical role in mediating tumor cell chemoresistance by promoting autophagy, modulating apoptotic pathways, and facilitating immune evasion. By generating NADPH and inducing autophagy, FAO reduces ROS levels and suppresses apoptosis [54,129]. In chemoresistant breast cancer models, enhanced FAO was shown to activate STAT3 via acetylation, which upregulates long-chain acyl-CoA synthetase 4 and subsequently drives phospholipid biosynthesis. These newly synthesized phospholipids integrate into mitochondrial membranes, increasing their lipid content, structural integrity, and membrane potential, ultimately conferring greater resistance to apoptosis [130].

In their study, Ye et al. demonstrated that leukemia cells exhibit higher rates of FAO compared to non-leukemia cells, with LSCs (Lin-) showing elevated FAO relative to more differentiated leukemia cells (Lin+). In a murine BC-CML co-culture model with adipocytes, a subpopulation of LSCs utilized gonadal adipose tissue (GAT) as a metabolic niche, suggesting that FAO regulation varies across LSC subpopulations. Additionally, targeting mitochondrial carnitine palmitoyltransferase-1 (CPT1) with etomoxir reduced FAO by approximately 60%, indicating that peroxisomal FAO makes a substantial contribution to total FAO in CML LSCs [15].

Several studies report that FAO is activated and supported by BM adipocytes. In an in vitro study of acute monocytic leukemia (AMoL) cells under nutrient starvation, co-culture with BM adipocytes increased malignant cell viability to a greater extent than co-culture with mesenchymal stem cells (MSCs). Mechanistically, this protection was driven, at least in part, by the upregulation of *Bcl2* mRNA and the induction of a heat shock protein (HSP) chaperone response [61]. These findings indicate that FAO in malignant cells is activated by co-culture with BM adipocytes. Correspondingly, ketone body levels, produced by FAO, were higher in co-culture media than in control media. Furthermore, a significant upregulation of HADHA, a crucial component of the mitochondrial trifunctional protein (MTP) catalyzing the final steps of FAO, confirmed that BM adipocytes stimulate FAO in AMoL cells. Crucially, these alterations in lipid metabolism are required for the anti-apoptotic effects observed in this system, as FAO inhibition using a selective CPT1 inhibitor completely abolished adipocyte-mediated protection [56,61].

Co-culture with BM adipocytes upregulated the stress-response kinases p-AMPK (AMP-activated protein kinase) and p-p38 MAPK alongside autophagy, while downregulating Akt/mTOR signaling via p-Akt depletion. Concurrently, elevated levels of major aerobic glycolysis products were detected, suggesting the activation of anaplerotic pathways that support mitochondrial FAO [61,131].

Gene expression analysis revealed elevated levels of lipid metabolism proteins in AMoL cells compared to control cells, including PPARγ, FABP4, CD36, and CPT1. Once activated, PPARγ upregulated its downstream target genes, such as FABP4, CD36, and BCL2 [61,132]. PPARγ is a nuclear receptor that regulates glucose and lipid metabolism and serves as a master regulator of adipogenesis [133]. Additionally, upregulation of the adiponectin receptor 1 (ADIPOR1) gene in AMoL cells further promoted AMPK pathway activation, complementing the finding that BM adipocytes express and secrete significantly higher levels of adiponectin than parental MSCs [61].

**Figure 2:**
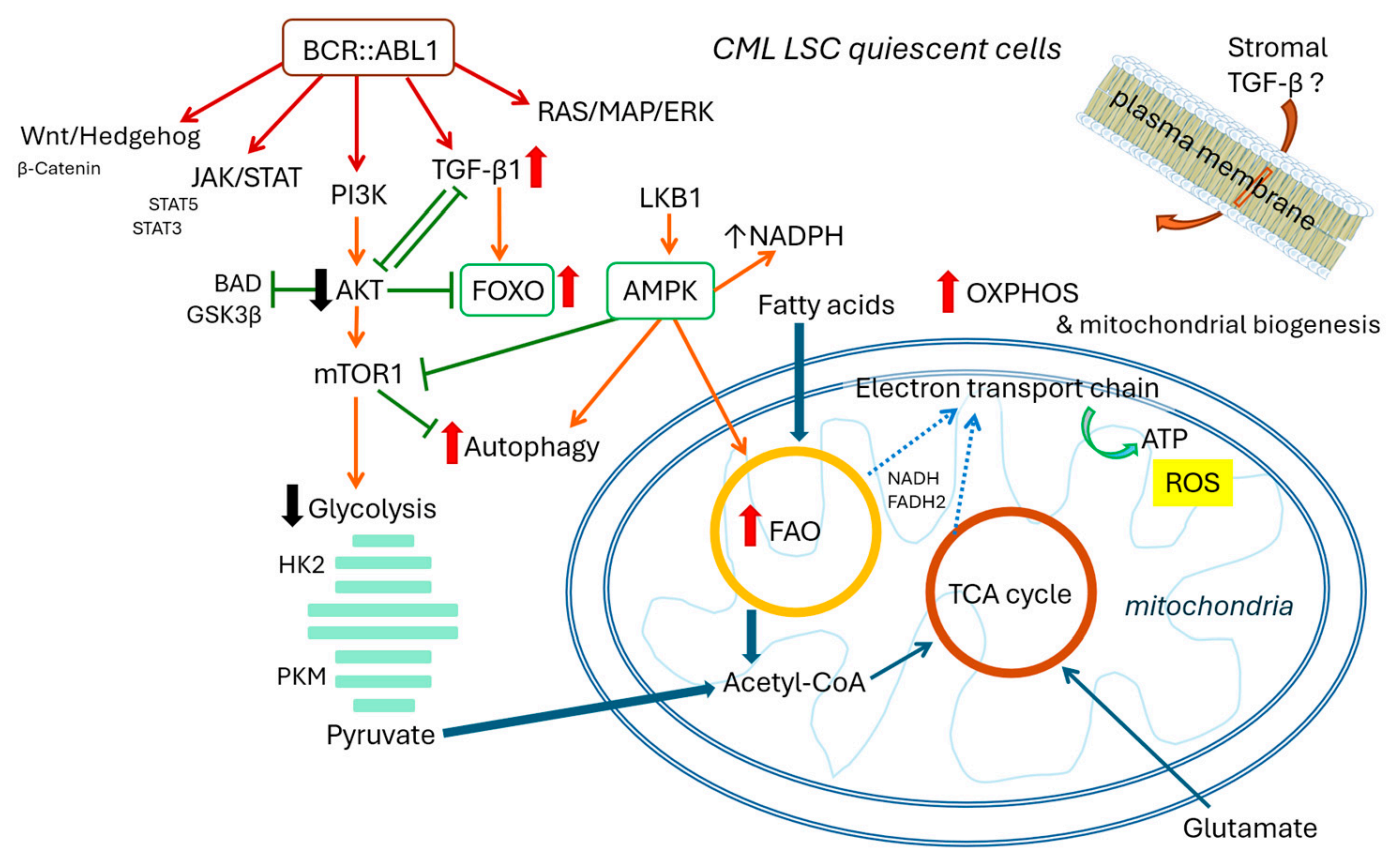
Schematic overview of signaling and metabolic pathways in quiescent CML LSCs. Although the BCR::ABL1 oncoprotein initiates several signaling cascades (red arrows), quiescent CML LSCs remain highly insensitive to these actions. PI3K/Akt signaling is downregulated, suppressing glucose uptake and glycolysis. Instead, the TGF-β/FOXO signaling pathway is critical for maintaining LSC self-renewal; TGF-β reduces Akt phosphorylation, enabling FOXO3a nuclear localization to drive the transcription of its target genes, including those involved in autophagy. Stromal TGF-β sources can influence LSC metabolism. Furthermore, CML LSC survival relies on elevated OXPHOS and fatty acid metabolism, with FAO supporting antioxidant regeneration and inhibiting apoptosis. CML LSCs display significantly higher autophagic flux and AMPK signaling pathway activity. Red arrows show direct stimulation by the oncogene. Orange arrows follow intracellular stimulating actions, while green T-headed arrows reflect a blocking effect. Blue arrows depict stimulating signaling in metabolic pathways and mitochondria. Light blue lines below glycolysis represent glycolytic enzymes; HK2 and PKM denote hexokinase 2 and pyruvate kinase muscle isoform, respectively. Bright red arrows show upregulated processes, while black arrows show downregulated processes.

These findings demonstrate the downregulation of several genes, including HK2, AGTRL1 (apelin receptor), ghrelin, CCL2 (MCP-1), PPP3R1 (calcineurin B), and TP53 (p53). Notably, AGTRL1 regulates glucose and lipid metabolism by enhancing glucose uptake while suppressing adipogenesis and lipolysis [61,134,135,136]. Overall, this study demonstrates a metabolic shift toward enhanced fatty acid uptake, FAO, and AMPK pathway activation, coupled with the downregulation of pathways supporting glucose uptake and glycolysis, such as PI3K/Akt signaling and its downstream targets (Figure 2).

#### 2.2.3. Lysophospholipids in CML

Beyond serving merely as energy sources, lipids play a far broader role in cancer pathogenesis. They act as bioactive signaling molecules—such as sphingosine-1-phosphate (S1P), PGE2, lipoxin, and ceramide—and actively participate in epigenetic modifications through fatty acylation [137,138,139]. Proper lysophospholipid (LysoPL) metabolism is required for the maintenance of CML LSCs in vivo. The LysoPL pathway produces lipid second messengers that mediate regulatory signaling in an oncogene-independent manner [10,133].

Researchers demonstrated that the Gdpd3 gene, which encodes a lysophospholipase D enzyme, is highly expressed in murine CML LSCs compared to normal stem cells. Lysophospholipase D is an ER membrane-associated enzyme that converts LysoPL to lysophosphatidic acids (LPAs) within the glycerol phosphate pathway. Disruption of this enzyme altered the levels of specific LPAs and lipid mediators in CML stem cells. Furthermore, Gdpd3 deficiency impaired the self-renewal capacity of CML LSCs and attenuated their ability to cause disease relapse [10,133]. Moreover, mice overexpressing human GDPD3 in the liver showed significantly increased hepatic LPA production and elevated FA uptake compared to control animals, but without affecting de novo FA synthesis, triglyceride content, or normal circulating lipid concentrations [131].

Specifically, Gdpd3 acts as a key suppressor of the AKT/mTORC1 pathway in CML stem cells. In Gdpd3-deficient cells, hyperactivation of this pathway disrupts LSC quiescence. By inhibiting Akt, GDPD3 promotes the nuclear localization and transcriptional activity of Foxo3a, as well as the nuclear interaction between Foxo3a and β-catenin [10,12]. Previous studies have established that Wnt/β-catenin signaling is required for the self-renewal of both normal and neoplastic stem cells [13,61]. Furthermore, the levels of prostaglandins, eicosanoids, and docosanoids were decreased in Gdpd3-deficient CML cells. Overall, these findings highlight GDPD3 as a primary repressor of proliferation and a critical driver of LSC self-renewal [10].

Beyond LysoPL and PPARγ signaling, other lipid-related enzymes—such as arachidonate 5-lipoxygenase (Alox5) and arachidonate 15-lipoxygenase (Alox15)—are critical for CML LSC survival. These targets have also been proposed for combination therapy to overcome resistance to TKIs [10,140].

#### 2.2.4. The AMPK Signaling and Autophagy in CML

AMPK signaling acts as a central cellular energy sensor. It is activated by metabolic stress, low intracellular energy levels (low ATP), ER stress, hypoxia, ROS, and stimulation of the adiponectin receptor, among other factors. During energy stress (glucose-deficient conditions), the Liver Kinase B1 (LKB1)–AMPK cascade promotes mitochondrial respiration (OXPHOS) and lipid utilization (FAO). AMPK regulates metabolic pathways by inhibiting mTOR (forming a negative feedback loop), suppressing ATP-consuming processes, and inducing AMPK-dependent cytoprotective autophagy and mitochondrial biogenesis [55,59,60,61]. Upon activation, AMPK increases FAO while suppressing FA, sterol, and glycogen synthesis. It enhances TCA cycle capacity, including anaplerosis, while maintaining NADPH levels in tumor cells. Mechanistically, AMPK promotes FAO by stimulating the phosphorylation of acetyl-CoA carboxylase (ACC), thereby inhibiting ACC and reducing malonyl-CoA—an allosteric inhibitor of CPT1A and CPT1B [56,141]. Additionally, the LKB1–AMPK axis counteracts oxidative stress caused by impaired glycolysis or high OXPHOS, protecting cancer cells from apoptosis during energy deprivation [54,58,60]. However, under severe ATP depletion, AMPK can also initiate cell death pathways [55,106,142].

According to some studies, AMPK activators may exert anti-leukemic effects against Ph+ leukemic cells, especially when combined with TKIs and autophagy inhibitors [106,142,143]. As a crucial cellular adaptation, autophagy is triggered by impaired energy metabolism, subsequently supporting mitochondrial activity to overcome energy imbalance. Coupled with heightened mitochondrial function, autophagy serves as an essential process for LSC quiescence and survival. Autophagy often acts as a protective mechanism in tumor cells, including imatinib-treated BCR::ABL1-expressing cells and the CD34+ 38− stem/progenitor cell population; notably, CD34+ CML LSCs display significantly higher autophagic flux compared to normal HSCs [55,58,144,145,146].

The BCR::ABL1/PI3K/Akt/mTOR signaling typically conveys autophagy inhibition through several mechanisms, including ATF5-mediated regulation of mTOR transcription, induction of miR-30a expression, and downregulation of key autophagy genes. It is hypothesized that autophagy is suppressed primarily to enable and conserve biosynthetic processes for cell proliferation and to prevent degradation of the oncoprotein. TKI treatment can restore autophagy and enable cell survival, thereby antagonizing the drug’s own proapoptotic effects. However, evidence also demonstrates that autophagy is essential for BCR::ABL1-dependent leukemogenesis. Although the oncogene suppresses autophagy, leukemic cells remain heavily dependent on the pathway to survive severe cellular stresses, such as nutrient or growth factor deprivation. Beyond metabolic status, autophagy is dictated by dominant signaling cascades, genetic profiles, cell types, and tumor stages. Furthermore, autophagy can exploit distinct mechanisms to modulate cellular stress, which will be discussed in the following sections [4,28,38,41,55,131,145,147,148].

In a study using K562 cells with active BCR::ABL1 signaling (and therefore not LSCs), continuous imatinib treatment inhibited glycolytic activity and induced autophagy, as evidenced by AMPK pathway activation. These cells subsequently relied on mitochondrial functions to maintain viability. Surprisingly, prolonged exposure to these conditions increased the sensitivity of CML cell lines to TKI-induced cell death. Indeed, imatinib-treated cells exhibited higher mitochondrial activity, elevated mitochondrial membrane potential (MMP), and increased cell death upon treatment with an ATP-synthase inhibitor [106]. Perhaps, despite FAO’s association with cancer progression and chemoresistance, high FAO activity ultimately exhausts FA pools and suppresses de novo phospholipid biogenesis [130], in the absence of microenvironmental lipid salvage.

The upregulation of mitochondrial biogenesis-related genes is observed in many OXPHOS-dependent cancers. CML LSCs exhibit elevated mitochondrial biogenesis—characterized by increased mitochondrial number, mass, and membrane potential (MMP)—features critical for their survival. Higher MMP is associated with altered electron transport and NAD+ regeneration, leading to a shift in the NAD+/NADH ratio. Furthermore, AMPK contributes to the replenishment of NADPH levels by stimulating the PPP [54,55,63,149].

To counteract BCR::ABL1-independent resistance, several studies propose targeting autophagy in combination with TKIs or mTOR inhibitors as a novel therapeutic strategy in CML [28,150,151]. Autophagy protects CML stem cells following TKI administration and may contribute to disease persistence. Imatinib has been shown to induce autophagy in various CML cell types through ER stress and intracellular calcium depletion. This autophagic response is independent of BCR::ABL1 activity and uncoupled from imatinib-induced apoptosis. Importantly, suppressing autophagy—such as through the knockdown of autophagy-related genes (ATG5 and ATG7)—enhances TKI efficacy and can substantially eliminate CML stem cells [60,145,148].

Activation of the ULK1 serine/threonine kinase complex represents an initiation step of autophagy under stress, connecting upstream energy sensors (such as mTOR) to downstream autophagosome assembly. Inhibiting ULK1 in combination with TKIs selectively targets CML LSCs, leading to a loss of quiescence, elevated oxidative stress, induction of differentiation, and resensitization to TKIs [146,152,153].

Moreover, the significance of autophagy is particularly evident in cells with acquired TKI resistance. Imatinib-induced autophagy was associated with reduced ROS levels—exerting a seemingly cytoprotective effect—as well as elevated intracellular glutamate levels. Findings indicate that acquired TKI resistance relies heavily on glutamine and other amino acid metabolic pathways. Specifically, high glutamine consumption and glutamate production drive self-protective mechanisms that boost proline synthesis to support proliferation, alongside glutathione metabolism for ROS scavenging. These and similar autophagy-related mechanisms of oxidative stress modulation (e.g., the KEAP1–NRF2 axis and PGC-1α-dependent antioxidant response) appear to protect mitochondria from degradation in LSCs [106,127,154]. The interplay between elevated ROS levels, AMPK activation, and autophagy is thought to keep ROS below the threshold that would otherwise trigger LSC differentiation [55].

Autophagy is also tightly linked to TGF-β and FOXO signaling, aligning with AMPK activity under metabolic stress. TGF-β promotes the FOXO3a-mediated transcription of autophagy genes [55]. Similarly, AMPK phosphorylates FOXO3a to drive the expression of mitochondrial, autophagy-related (BECN1, MAP1LC3B), and antioxidant genes (e.g., thioredoxin). Conversely, the loss of FOXO activity markedly increases ROS levels [5,94,155,156].

The ubiquitin-proteasome pathway and autophagy-related mechanisms are also being investigated as targeted therapeutic interventions in CML. One example is disulfiram, which synergizes with TKIs to target resistant cells and LSCs [104]. A study by Zeng and colleagues showed that disulfiram destabilizes glutathione peroxidase 4 (GPX4) by binding to HSPA8, thereby promoting GPX4 ubiquitination and degradation. This loss of GPX4 intensifies lipid peroxidation, triggering ferroptosis and subsequently eradicating TKI-insensitive LSCs. Further delineation of the mechanisms targeting this regulatory network could profoundly impact tumor biology.

#### 2.2.5. The PPAR-γ Coactivator (PGC)-1α, Autophagy and Oxidative Stress

The transcription co-activator PGC-1α is another major player in the homeostasis of energy metabolism, influencing mitochondrial function, biogenesis, and oxygen consumption rate. PGC-1α expression and activity are regulated by multiple factors, including extracellular stimuli, cellular stressors, hormones, and cytokines. For instance, the NF-κB, MAPK, or Akt signaling pathways can modulate PGC-1α transcription, whereas AMPK, MAPK, Akt, and S6 kinase are involved in its post-translational regulation [26,54,66,157,158,159,160,161].

In CML, PGC-1α is upregulated in stem/progenitor cells and plays an integrative role in metabolic fitness, thereby supporting the viability of leukemia cells. Consequently, it enhances mitochondrial activity and increases OXPHOS, helping cells evade apoptosis by promoting ATP production during bioenergetic stress [161]. High PGC-lα expression has also been linked to TKI resistance in CML stem/progenitor cells, whereas its inhibition sensitizes cells to apoptosis when combined with nilotinib. However, PGC-lα may play a dual role in malignancies, often depending on tissue specificity [26,54,66,157,158,159,160].

The AMPK/PGC-1α axis regulates cellular energy balance. AMPK increases the transcriptional activity of PGC-1α, which is required for the expression of AMPK-induced genes, including those mediating mitochondrial function [161]. Additionally, as mentioned, AMPK activates cytoprotective autophagy while suppressing ATP-consuming processes and modulating ROS levels. Thereby, this signaling network serves to maintain energy homeostasis and protect leukemia cells from apoptotic stimuli [127,149,162]. Nevertheless, in situations of prolonged oxidative stress or when ATP levels are sufficiently low, this energy-guided signaling network is disrupted. Consequently, different outcomes may arise, such as ROS-induced differentiation of CML LSCs or sustained AMPK activation, which can initiate regulated cell death pathways. In addition, prolonged overproduction of ROS inhibits autophagy and decreases PGC-1α activity [54,55,142,155].

PGC-1α serves as an important regulator of ROS-scavenging mechanisms; it is activated under oxidative stress and responds by upregulating the antioxidant defense system. However, evidence indicates that PGC-1α and NF-κB are reciprocally regulated. Specifically, during inflammation, NF-κB activation and the associated increase in ROS suppress PGC-1α transcription; conversely, diminished PGC-1α levels and the resulting loss of antioxidative support promote NF-κB activity [161,163].

The relationship between PGC-1α and autophagy operates in a dynamic, context-dependent manner. In the presence of high FFA levels, PGC-1α gene repression occurs through non-CpG methylation of its promoter as a form of metabolic adaptation to downregulate mitochondrial biogenesis and density. In their study, Barres et al. also demonstrate the same effects after exposure to inflammatory cytokines (e.g., TNFα) using primary human myocytes [164]. In contrast, both saturated and unsaturated FAs can trigger autophagy, albeit via distinct molecular mechanisms [165]. Fatty acid-induced autophagy serves as an important pro-survival mechanism in CML. For instance, a study on autophagic cell death in the K562 cell line highlighted the impact of the FA derivative AIC-47 (a 3-decenoic acid analogue) and its modulation of the PKM isoform expression profile. The authors observed that forcing a switch from PKM2 to PKM1 expression compels cells to rely on OXPHOS, which subsequently triggers autophagic cell death by elevating ROS levels. Furthermore, AIC-47-mediated repression of BCR::ABL1 signaling occurred via the PPARγ/β-catenin pathway and the downregulation of c-Myc. This delineates a clear mechanism by which FAs modulate autophagic flux in BCR::ABL1-positive cells [41].

However, studies also show discrepancies in cellular responses to FAs depending on experimental design and timing. Palmitate can either stimulate or inhibit AMPK signaling, depending on the cell type and concentration [165,166]. A comparative study evaluating the cellular effects of multiple FAs, including parameters linked to autophagy, demonstrated that *trans*-unsaturated FAs were less active in stimulating a stress response than their *cis*-isomers and were unable to stimulate an autophagic response. Furthermore, several *trans*-unsaturated FAs were highly efficient at suppressing the autophagy and ER stress induced by palmitic acid in cultured human cells [167].

#### 2.2.6. Sirtuins in the Regulation of Metabolic Signaling in CML

Silent information regulators (sirtuins, SIRTs) are NAD+ -dependent deacetylases and signaling proteins involved in critical biological processes. They act as stress sensors that regulate the cell cycle, maintain redox balance, and enhance mitochondrial function. Decreased glycolytic flux and associated alterations in the NAD+/NADH ratio activate sirtuins. A key regulatory event is mediated by SIRT1, which deacetylates PGC-1α in an NAD-dependent manner, reflecting the cellular energy status. Beyond PGC-1α, sirtuin target proteins include p53, NF-κB, FOXOs, HMGCS2, superoxide dismutase 2, structural proteins, and the DNA repair enzyme Ku70 [26,67,94,160,168,169].

SIRT1-mediated deacetylation is a pivotal post-translational modification that fine-tunes FOXO3a activity. Under oxidative stress, SIRT1 decreases the expression of the pro-apoptotic protein Bim via FOXO3a—thereby preventing cell death—while upregulating antioxidant enzymes (e.g., MnSOD) and cell-cycle arrest genes (e.g., cyclin-dependent kinase inhibitor 1B). Working alongside SIRT2, SIRT1 enables cells to repair DNA damage prior to division [26,94,95,96]. Nevertheless, hyperactivated SIRT1 in CML cells has been reported to impair DNA repair fidelity, thereby promoting the accumulation of genetic mutations that confer drug resistance [74].

SIRT1 overexpression in CML LSCs is regarded as a key survival mechanism activated downstream of BCR::ABL1/STAT5 signaling. STAT5 appears to directly upregulate the SIRT1 promoter; however, STAT5 knockdown only partially suppresses SIRT1 expression, indicating that SIRT1 activation is not entirely dependent on BCR::ABL1 kinase activity. Accordingly, dual targeting of SIRT1 and BCR::ABL1 kinase improved survival in an animal model of CML [64].

SIRT1 overexpression is involved in the mitochondrial metabolic rewiring associated with LSC maintenance and TKI resistance [26,65]. Studies show that the SIRT1/PGC-1α axis drives metabolic alterations—specifically a shift from glycolysis toward OXPHOS—in a colon cancer model [170]. Moreover, suppressing the SIRT1/PGC-1α/NRF2 pathway increases the susceptibility of wild-type p53 cancer cells to oxidative stress and apoptosis [171]. Furthermore, SIRT1 overexpression correlates with enhanced conversion of LC3, a key ubiquitin-like protein essential for autophagosome formation, in K562 cells [67].

Sirtuins regulate the expression of genes involved in FA metabolism, thereby supporting both FAO and OXPHOS. Specifically, SIRT1 is associated with distinct OXPHOS gene expression signatures [26]. Furthermore, key enzymes such as CPT1A and long-chain acyl-CoA dehydrogenase (LCAD) are directly activated through sirtuin-mediated deacetylation [56,172]. Knockdown and transgenic mouse experiments demonstrate that the SIRT1/PGC-1α axis is a critical contributor to enhanced OXPHOS capacity and TKI resistance in CML LSCs [26,64,66].

Using a transgenic CML mouse model, Abraham and colleagues observed that genetic deletion of SIRT1 significantly reduced basal and maximal mitochondrial respiration, as well as mitochondrial reserve capacity, compared with control CML c-Kit+ cells. Conversely, SIRT1-deleted CML cells exhibited no significant alteration in maximal glycolysis or glycolytic reserve. The inhibitory effects of SIRT1 deletion on mitochondrial function were not observed in normal hematopoietic stem/progenitor cells [26].

Interestingly, leucine supplementation increased SIRT1 expression and stabilized mitochondrial function in high-fat diet-induced obese mice. This was concordant with the deacetylation of PGC-1α and FOXO1, as well as the upregulation of genes controlling FAO. Ultimately, leucine attenuated mitochondrial dysfunction in obese mice [173]. It is also worth noting that SIRT3 plays a critical role in mitochondrial homeostasis by modulating major pathways involved in mitochondrial function and biogenesis, including FOXO3. In AML, SIRT3 has been shown to confer chemoresistance by regulating OXPHOS [27,111,174,175].

Overall, altered mitochondrial respiration with stimulated OXPHOS in CML LSCs aligns with SIRT1/PGC-1α axis-mediated adaptations in mitochondrial function and energy production. Collectively, these findings indicate that sirtuins represent potential therapeutic targets for overriding survival mechanisms and overcoming drug resistance in CML LSCs.

### 2.3. Involvement of BM Microenvironment in CML

Another important aspect in CML is the reciprocal interaction between leukemic cells and the BM microenvironment. The HSC niche comprises various cell types, including mesenchymal stem/stromal cells (MSCs), adipocytes, endothelial cells, fibroblasts, osteoblasts, and megakaryocytes. In adults, adipocytes occupy up to 70% of the total BM volume. The emergence of a CML clone alters both the cellular composition and molecular milieu of the HSC niche, creating a tumor-supportive microenvironment that sustains LSCs. Accumulating evidence suggests that TKI resistance is at least partly driven by this dysregulated BM microenvironment [8,78,124,176].

Numerous soluble factors—including cytokines, chemokines, and growth factors such as IL-1, IL-6, TNF-α, MIP-1α/β, MCP-1, and G-CSF—participate in this crosstalk, delivering signals that preferentially favor malignant over normal HSCs [8,78,79,85]. For example, TNF-α alters chemokine ligand and receptor expression in a murine CML model, thereby driving LSC proliferation and self-renewal. This pathway operates through the upregulation of CXCL1 in stromal progenitors and CXCR2 in LSCs, which directly supports the leukemic clone [8,76,77,78]. Furthermore, elevated IL-8 expression acts via autocrine and paracrine signaling, thereby facilitating LSC adhesion to MSCs and sustaining their survival [177].

Single-cell transcriptomic analyses have demonstrated a cell-extrinsic disruption of hematopoiesis in CML, revealing heterogeneity within the leukemic stem cell compartment—notably a distinct subpopulation that persists despite prolonged therapy [79]. Mesenchymal stromal cells exert immunoregulatory functions and protect CD34+ CML progenitors from imatinib via direct contact and paracrine signaling [13,91,92,93,137]. Specifically, the CXCL12 (SDF-1)/CXCR4 interaction between MSCs and CML cells protects leukemic cells from caspase-3-dependent apoptosis [173].

It has been reported that MSCs support quiescence in CML cell lines and can inhibit CML cell proliferation in vitro by producing high levels of IFN-α. MSCs prevent apoptosis in CML cells by downregulating Cyclin D2, caspase-3, and Bax; modulating the expression of apoptosis-related proteins such as Bcl-2; secreting IL-7; and activating JAK1–STAT5 signaling in a BCR::ABL1-independent manner [36,37,91]. In the blast phase, MSCs enhance the anti-apoptotic capacity of CML cells by regulating apoptotic protein expression and activating the Wnt/β-catenin signaling pathway [13,36].

Interaction with stromal cells protected N-cadherin- and CD34-enriched CML cells from imatinib-induced apoptosis, maintaining them in a quiescent, non-cycling state characterized by elevated phosphorylation of ERK1/2 MAPK (via the actin cytoskeleton) and SMAD1/8. Notably, prolonged stromal contact induced persistent TKI resistance even after detachment from the stroma [13,16,178]. In contrast, Jalilivand et al. showed that healthy donor BM MSC-exosomes inhibit key signaling pathways in AML cells. Specifically, these exosomes downregulated JAK2, STAT3, and STAT5 expression—thereby blocking JAK/STAT signaling and suppressing leukemic growth. This effect was mediated by upregulation of ROS, p53, p21, BAX, and FOXO4, accompanied by concomitant reduction in BCL2 and c-Myc [17,179].

Tumor-associated adipocytes (TAAs) have been shown to support cancer cell proliferation and disease progression. This complex bidirectional dialogue is driven largely by cancer cells and is centered around lipid metabolism. Upon co-culture with cancer cells, adipocytes undergo significant structural and functional alterations. Additionally, these remodeled adipocytes secrete a variety of adipokines and exosomes, thereby reprogramming cancer cell metabolism [88,177,179,180].

Tumor-associated stromal cells actively exchange metabolites with malignant cells [99]. In leukemia, LSCs establish a close functional interplay with surrounding adipocytes. Specifically, BM adipocytes play a critical role in hematologic malignancies by secreting key bioactive factors, including adipokines and FFAs [15,89]. Research evidence indicates that leukemic blasts expand within an adipocyte-rich environment in the BM, where AML and BP-CML cells exploit nearby adipocytes to meet their bioenergetic and biosynthetic demands. Adipocytes serve as a critical energy source by releasing FAs for use by LSCs, thereby supporting their viability. Consequently, these LSCs exhibit enhanced TCA cycle and FAO, accompanied by broad TCA substrate utilization [3,15,131,177].

AML blasts induce metabolic remodeling in TAAs via activation of hormone-sensitive lipase and transcriptional upregulation of the lipid chaperone FABP4, significantly enhancing intercellular FA transport. Adipocytes integrate FABP4 into the plasma membrane and release it extracellularly, enabling leukemic cells to take up FABP4 and accelerate FA internalization. Furthermore, FABP4 drives additional non-transport signaling events, including the upregulation of FA transporters such as CD36 (fatty acid translocase). CD36 promotes FA uptake and acts as a downstream target of the IL-6/STAT3 signaling axis [177,180,181,182].

Activation of lipolysis in adipocytes releases FAs that further promote STAT3 phosphorylation, thereby inducing its non-genomic regulatory effects on lipid metabolism. STAT3 inhibits enzymes in the glycerolipid synthesis pathway involved in TG synthesis. In this manner, STAT3 dictates the metabolic fate of FAs within adipocytes without altering the rate of lipolysis [182,183,184].

In a mouse model of blast crisis CML, Ye et al. identified a distinct CD36+ LSC subpopulation harboring a supportive, drug-resistant niche within gonadal adipose tissue. These LSCs stimulated adipocyte lipolysis to fuel their own FAO—a trait particularly pronounced in CD36+ relative to CD36- LSCs, and one that was selectively blocked by CD36 inhibition. Gonadal adipose tissue containing LSCs released significantly higher levels of free FAs and FABP4 than control tissue. Phenotypically, LSCs adopted a pro-inflammatory profile characterized by marked upregulation of IL-1, IL-6, TNF-α, CXCL1–3, and GM-CSF. Notably, CD36+ LSCs maintained lower ATP levels than glycolysis-dependent CD36- LSCs and demonstrated heightened resistance to Complex I inhibition. This CD36+ subset exhibited relative quiescence, marked by elevated cell cycle inhibitors (e.g., Foxo1) and reduced expression of cell cycle drivers like Myc [15].

Adipocytes are a key source of adipokines, including pro-inflammatory cytokines, chemokines, and angiogenic factors such as IL-6, TNF-α, IL-1, MCP-1, CCL5, MMP-11, and VEGF. Pro-inflammatory cytokines secreted by adipocytes may partially drive FA release [89,90,185,186].

#### Exosomes in CML

Exosomes present a vital means of crosstalk between leukemic and stromal cells. In vitro and in vivo studies report that CML-derived exosomes promote the proliferation and survival of the malignant clone through several mechanisms. An exosome-mediated autocrine loop was demonstrated for CML-derived exosome-associated TGF-β1, leading to the downstream activation of ERK/Akt/NF-κB signaling and anti-apoptotic pathways. Specifically, exosome-targeted CML cells (LAMA84 cell line) demonstrated increased levels of anti-apoptotic proteins (Bcl-w, Bcl-xL, and survivin) and reduced levels of pro-apoptotic proteins (Bad, Bax, and Puma). Inhibition of the TGF-β1 receptor significantly reversed these exosome-mediated changes. This autocrine loop is further supported by evidence that the BCR::ABL1 oncogene upregulates TGF-β1 expression [18,19,187]. Importantly, exposure of the K562 leukemia cell line to a TKI significantly reduced total exosome release [20,21].

Wei et al. demonstrated across multiple cell types that tumor cell exosome secretion is governed by PKM2, showing a positive correlation between exosome release and aerobic glycolysis. Specifically, PKM2 phosphorylates synaptosome-associated protein 23 (SNAP-23), promoting SNARE complex assembly (which mediates membrane fusion) and driving exosomal exocytosis. This secretory pathway relies heavily on robust aerobic glycolysis, with exosome output scaling directly with PKM2 levels. Furthermore, exosomal membranes harbor various TNF superfamily members, and TNF-α-driven aerobic glycolysis accelerates exosome release from tumor cells [9,188]. Fatty acid oxidation, which is vital for LSC survival, was also shown to contribute to extracellular vesicle biogenesis in normal HSCs through NADPH production [189].

Corrado et al. demonstrated that CML-derived exosomes (LAMA84 cell line) modulate the BM microenvironment via activation of the EGF receptor on stromal cells (HS5 and primary BM stromal cells). This activation is mediated by exosomal amphiregulin (AREG, an EGF family ligand) binding to the EGF receptor on stromal cells, which triggers signaling that upregulates the SNAIL transcription factor and its downstream targets, MMP-9 and IL-8, thereby driving CML cell proliferation. Additionally, CML exosomes induce annexin A2 expression, enhancing the adhesion of CML cells to the stromal monolayer [177,190,191].

Importantly, AREG is produced by genotoxically damaged stromal cells that have entered senescence. Via paracrine signaling, AREG induces programmed cell death 1 ligand (PD-L1) expression in cancer cells, thereby impairing anti-tumor immune clearance. AREG also sustains the suppressive function of Tregs through the EGFR/GSK-3β/Foxp3 signaling axis. Ultimately, these mechanisms drive acquired treatment resistance and promote cancer progression [186,192].

## 3. Conclusions

A growing body of evidence reveals numerous novel, BCR::ABL1 kinase-independent survival mechanisms in CML stem/progenitor cells that could serve as therapeutic targets. Current findings highlight substantial metabolic reprogramming in LSCs that profoundly affects their survival. Indeed, alterations in energy metabolism—specifically elevated FAO and enhanced OXPHOS, which are inseparable from mitochondrial function—constitute a distinct metabolic hallmark of CML LSCs. This metabolic network in CML is closely regulated by an interplay of intracellular, extracellular, oncogenic, and physiological signals and drives the adaptation of LSCs to their microenvironment.

## 4. Implications for Patient Care and Future Directions

Metabolomic profiling and bioanalysis reveal pronounced metabolic alterations in CML patients, particularly within energy and nucleotide pathways [192]. Several studies have evaluated metabolite levels in the blood plasma and leukocytes of CML patients in relation to TKI response and overall prognosis. For instance, myristic acid, d-sorbitol, and glycerol have emerged as potential biomarkers for TKI responsiveness, while glutamate, hypoxanthine, and D-galactonic acid were key metabolites differentiating patients who achieved a deep molecular response from those with suboptimal outcomes. Overall metabolic profiles clearly distinguished newly diagnosed CML patients from those receiving TKI therapy, with alterations being more pronounced in leukocytes than in patient plasma. Newly diagnosed patients show increased intracellular levels of phosphorylated hexoses in leukocytes alongside decreased plasma hexose levels—a shift attributed to hyperactivation of the PI3K pathway. TKI treatment reversed these alterations, reducing leukocyte levels of phosphorylated hexoses while restoring plasma levels to control values. Additionally, newly diagnosed patients exhibited a decline in 3-phosphoglycerate and most TCA cycle intermediates relative to the TKI-treated group. Consequently, TKI-mediated suppression normalized TCA cycle parameters [107,110,128,192].

Unlike the majority of amino acids, which were elevated compared to controls, aspartate levels were markedly reduced. Furthermore, distinct differences in amino acid and acylcarnitine profiles were observed between responders and non-responders to TKI therapy. A markedly different metabolic signature was also associated with dasatinib treatment compared to imatinib or nilotinib. Broadly, newly diagnosed CML patients exhibit pronounced systemic metabolic disruptions that tend to normalize following TKI therapy [107,110,128,192].

A study evaluating a panel of metabolic biomarkers in CML identified several differentially expressed metabolites compared to healthy controls. Newly diagnosed CML patients exhibited elevated levels of lactic acid, carbohydrates, glycine, and isoleucine alongside decreased levels of FAs. Among the evaluated biomarkers, glycerol and myristic acid (a long-chain saturated FFA) showed the strongest association with TKI response. Furthermore, TKI treatment effectively restored the metabolic profile toward healthy control levels in TKI-sensitive patients achieving optimal therapeutic responses, unlike in TKI-resistant individuals (Table 4) [107].

Of note, dasatinib was observed as a potent inducer of PGC-1α expression across various adipocyte subtypes in both lean and obese mice. Elevated hepatic PGC-1α levels upregulated gluconeogenic gene expression, triggering glucose intolerance in obese, but not lean, mice. This PGC-1α induction is proposed to stem from dasatinib-mediated inhibition of multiple off-target kinases beyond BCR::ABL1. Therefore, glucose homeostasis should be closely monitored in obese and diabetic CML patients receiving dasatinib [27].

Levels of the branched-chain amino acid isoleucine were significantly higher in the plasma of CML patients compared to healthy controls. Beyond protein synthesis, this amino acid participates in signaling pathways and glucose metabolism [107]. A significant limitation of measuring metabolites as circulating biomarkers in CML, and in general, is that numerous confounding factors can influence blood levels, including nutrition, medications, and chronic diseases [107,110,128].

The findings of this review underscore the formidable complexity of metabolic and signaling pathways, complicating efforts to elucidate the pathophysiological mechanisms underlying LSC survival. To address this challenge, recent advancements have focused on combination therapies to overcome LSC resistance. Abundant evidence indicates that targeting metabolic vulnerabilities alongside TKIs represents a promising therapeutic strategy for improving CML patient outcomes. Moving forward, future research directions center on utilizing cytokines as therapeutic targets or biomarkers, alongside disrupting protective LSC niches within the BM microenvironment.

Quiescent LSCs survive by rewiring their energy metabolism, depending heavily on mitochondrial OXPHOS and FAO. Moreover, emerging studies emphasize the significance of autophagy and redox balance within an intricate network that controls energy metabolism in leukemia cells. Therefore, further investigations are needed to decipher the links among autophagy, the AMPK/SIRT/FOXO/PGC-1α signaling cascade, and LSC maintenance. A deeper understanding of these associations will guide the future development of more effective anti-leukemic strategies to counteract CML LSC survival.

## Figures and Tables

**Table 1:** Summary of the baseline cellular characteristics in CML clone.

Cellular Phase	Predominant Cellular Components	Cytogenetic Characteristics	Underlying Pathophysiological Mechanisms	Proliferating Cells vs. Leukemia Stem Cells (LSCs)	Current Treatment Strategies	Mechanisms of Therapeutic Resistance	References
**Chronic Phase (CP)**	Amplification of mature and maturing myeloid cells (neutrophils, eosinophils, basophils) and blasts (<10%).	Presence of the Philadelphia chromosome (Ph+) in all dividing cells.	Constitutive activation of the BCR::ABL1 tyrosine kinase, leading to unregulated cell proliferation and reduced apoptosis.	**Proliferating** CML cells (progenitor & precursor cells) make up most of the CML clone. These are highly sensitive to BCR::ABL1 targeted therapies like TKIs. **LSCs** are the rare, quiescent disease-initiating pluripotent cells with unlimited self-renewal capacity, and remain BCR::ABL1-independent.	**First-line**: Tyrosine Kinase Inhibitors (TKIs) like Imatinib, Dasatinib, or Nilotinib. Interferon-alpha and hydroxyurea can also be used. Standard TKI treatment fails to eradicate LCSs.	**TKI Resistance**: BCR::ABL1 kinase domain mutations (e.g., T315I), clonal evolution, and intrinsic LSC quiescence. Interaction with stromal cells protects CD34+-enriched CML cells from imatinib-induced apoptosis, maintaining them in a quiescent state with elevated activity of ERK1/2 MAPK. LSCs induce lipolysis in adipocytes and use FA for their FAO. An exosome-mediated autocrine loop in CML-derived exosome-associated TGF-β1 activates anti-apoptotic pathways.	[1,2,3,4,5,6,7,13,14,15,16,17,18,19,20,21]
**Accelerated Phase (AP)**	Mounting burden of immature progenitors, worsening anemia, and fluctuating platelet counts. Blasts (10–19%) in blood/marrow; ≥ 20% basophils in peripheral blood.	Ph+ with additional clonal chromosomal abnormalities in Ph+ cells, such as trisomy 8, trisomy 19, isochromosome 17, monosomy 7/7q deletion, and variant translocations.	Disease acceleration driven by accumulating genetic/epigenetic alterations and genomic instability. Acquired mutations or the aberrant activation of pathways downstream of BCR::ABL1 kinase activity.	**Proliferating cells** include a higher burden of blast-committed progenitors. **LSCs** acquire further genetic mutations, driving the transformation and expansion of more primitive stem-like clones.	**Treatment**: Second-generation or third-generation TKIs (e.g., Ponatinib), sometimes followed by allogeneic hematopoietic stem cell transplantation (HSCT).	**Acquired kinase** domain mutations that evade TKI binding, drug efflux pumps, and upregulation of alternative survival signaling pathways (e.g., WNT/β-catenin). IL-6/JAK-STAT3 activation driven by external stimuli, independent of BCR::ABL1, may contribute to TKI resistance. SIRT1 overexpression-driven mitochondrial metabolic rewiring associated with LSC maintenance and TKI resistance.	[1,2,3,4,5,6,7,13,14,22,23,24,25,26,27]
**Blast Phase (Blast Crisis)**	Rapid proliferation of immature blasts, predominantly of myeloid origin, resembling acute leukemia. More than 20% blasts in BM; extramedullary blast proliferation.	Complex karyotypes with multiple additional chromosomal abnormalities; behaves similarly to acute leukemia. Reduced telomere length and telomerase activity.	Arrest in cell differentiation, massive accumulation of immature blasts, and activation of alternative pathways (e.g., WNT/β-catenin). Inefficient DNA damage repair mechanisms. TP53 mutations. IKZF1 deletion. Loss or dysfunction of essential transcription factor C/EBPα, including its post-transcriptional modification by the hnRNP E2 RNA-binding protein.	**Proliferating cells** are immature blasts. **LSCs** include committed Granulocyte-Macrophage Progenitors (GMPs) that have abnormally acquired stem-cell-like self-renewal capabilities.	**Treatment**: Intensive combination chemotherapy (like AML/ALL regimens) plus a TKI, followed by allogeneic HSCT if the patient achieves a second chronic phase. Various chemotherapy regimens with/without TKIs such as hypomethylating agent (HMA) plus venetoclax.	**Complete loss** of oncogene addiction, rapid emergence of compound mutations, and expanded stem cell fractions that are inherently drug-resistant. DNA damage repair and gene instability. Epigenetic dysregulation. Changes in drug influx/efflux pumps. Dysregulated interactions between hematopoietic and stromal cells. MSCs enhance the anti-apoptotic capacity of CML cells by regulating apoptotic protein expression.	[1,2,3,4,5,6,7,14,23,24,28,29,30,31,32,33,34,35,36,37]

FA—fatty acids, FAO—fatty acid oxidation, TP53—gene encoding the p53 protein, IKZF1—Ikaros encoding gene, MSCs—mesenchymal stem cells.

**Table 4:** Comprehensive summary of signaling and metabolic pathways, metabolic biomarkers, and their changes following TKI treatment in CML progenitor vs. stem cells.

	CML Progenitor Cells	Leukemia Stem Cells (LSCs)	References
Signaling pathways	The PI3K/Akt/mTOR signaling The BCR::ABL1/STAT5/SIRT1 axis The RAS/RAF/MAPK pathway The NF-κB pathway	The downregulation of PI3K/Akt signaling. The TGF-β/FOXO signaling The Liver Kinase B1 (LKB1)–AMPK cascade. The AMPK–FOXO3a axis The WNT/β-catenin pathway The Hedgehog (Hh, SHH/SMO) signaling The protein kinase C (PKC) pathway The SIRT1/PGC-1α axis. SIRT1 overexpression in CML LSCs is regarded as a key survival mechanism.	[13,26,40,42,61,94,95,97,111,134,135,136,155,156].
Metabolic pathways	The PI3K/Akt signaling supports glucose uptake and glycolysis. HK2 protects mitochondria from oxidative stress and promotes autophagy during glucose starvation. The PI3K/PKM2 axis diverts glycolytic intermediates toward anabolic pathways for the biosynthesis of amino acids, lipids, and nucleotides. PKM2 also plays a role in redox homeostasis. PKM2 knockdown reduces glycolysis, inhibits cell growth, and induces apoptosis.	CML LSC metabolism relies on increased OXPHOS and FA metabolism. FAO plays a crucial role in LSC self-renewal. FAO-derived acetyl-CoA is utilized to generate cytosolic NADPH, which supports antioxidant regeneration and suppresses ROS-induced apoptosis. FAO activates STAT3 via acetylation, leading to increased phospholipid biosynthesis and greater mitochondrial resistance to apoptosis. LSCs exhibit metabolic flexibility by utilizing diverse fuel sources, including glucose, fatty acids, amino acids, and anaplerotic substrates such as glutamate The AMPK–FOXO3a axis has been shown to regulate autophagy-related genes and antioxidant genes. AMPK suppresses ATP-consuming processes and stimulates PPP, replenishing NADPH. Glutamate and branched-chain amino acids pathways.	[3,10,11,26,40,41,55,60,85,114,115,116,117,118,124,130,134]
Key transcriptional regulators	BCR-ABL activity relies on STAT5, c-MYC, and NF-κB.	In CML LSC, key transcriptional regulators include: FOXO3a, β-catenin, GLI (of the Hh pathway), SIRT1, PPARγ, PGC-1α.	[10,23,33,34,38,46,61,75,101,132]
Metabolic biomarkers	Treatment-naïve (TN) patients show increased levels of phosphorylated hexoses in leukocytes alongside decreased plasma hexose levels. They exhibit elevated levels of lactic acid, carbohydrates, and glycine, alongside decreased levels of fatty acids. Myristic acid, glycerol, and d-sorbitol levels were reduced, while d-galactose, d-glucose, and myo-inositol were increased in TN patients. Increase in 3-phosphoglycerate. Increased levels of glutamate and malate. Aspartate levels are markedly reduced in TN patients. Levels of the branched-chain amino acid isoleucine were significantly higher in the plasma of CML patients.		[75,92,93,94,110,116,192]
Biomarker changes following TKI treatment	TKI treatment reduced leukocyte levels of phosphorylated hexoses, while restoring plasma levels to control values. Myristic acid, glycerol, and d-sorbitol show elevated levels in TKI-sensitive patients. D-galactose, d-glucose, and myo-inositol decreased following TKIs. Decrease in glycolytic intermediates including 3-phosphoglycerate in TKI-sensitive patients. Higher concentrations of malate, 5-aminoimidazole-4-carboxamide ribonucleotide (AICAR), hypoxanthine, D-galactonic acid, hippurate, citramalate, glutamate, xanthine, and 3-hydroxybenzoic acid were detected in those with suboptimal TKI response. Medium- and long-chain leukocyte acylcarnitines were reduced in non-responders to TKI therapy.		[75,92,93,94,110,116,192]

FAO—fatty acid oxidation, HK2—hexokinase 2, NADPH—nicotinamide adenine dinucleotide phosphate, PKM2—pyruvate kinase muscle type 2, PPP—pentose phosphate pathway, TCA—tricarboxylic acid, TN—treatment-naive.

## Data Availability

No new data were created or analyzed in this study.

## Abbreviations

The following abbreviations are used in this manuscript:

ACC	acetyl-CoA carboxylase
ADIPOR	adiponectin major receptor
AGTRL	apelin receptor
Akt	protein kinase B
ALDH	aldehyde dehydrogenase
ALL	acute lymphoblastic leukemia
Alox	arachidonate lipoxygenase
AML	acute myeloid leukemia
AMoL	acute monocytic leukemia
AMPK	AMP-activated protein kinase
AREG	amphiregulin
ATG	autophagy-related gene
ATM	ataxia-telangiectasia mutated
BAD	Bcl-2-associated death promoter
BCAA	branched-chain amino acid
Bcl-2	B-cell lymphoma 2
Bcl-6	B-cell lymphoma 6
BCL-X(L)	B-cell lymphoma-extra large
BCR::ABL1	breakpoint cluster region (BCR) and Abelson murine leukemia viral oncogene homolog 1 (ABL1)
BM	bone marrow
BMP	bone morphogenic protein
cAMP	cyclic adenosine monophosphate
CDKN1C	cyclin-dependent kinase inhibitor 1C
CIDEC	cell death-inducing DFF45-like effector CCD–cluster of differentiation
CML	chronic myeloid leukemia
CPT1	carnitine palmitoyltransferase 1
CXCL	chemokine C-X-C motif ligand
EGF	epidermal growth factor
ERK	extracellular signal-regulated kinase
ETC	electron transport chain
FA	fatty acid
FABP4	fatty acid-binding protein 4
FAO	fatty-acid oxidation
FOXO	forkhead box O
Gdpd3	Glycerophosphodiester Phosphodiesterase Domain Containing 3
GLUT	glucose transporter
GM-CSF	granulocyte-macrophage colony-stimulating factor
GSK3	glycogen synthase kinase 3
HIF-1α	hypoxia-inducible factor 1
HK	hexokinase
HSC	hematopoietic stem cells
HSP	heat shock protein
IFN	interferon
JAK	Janus kinase
KEAP1	Kelch-like ECH-associated protein 1
LCAD	long-chain acyl-CoA dehydrogenase
LEF-1	lymphoid enhancer-binding factor 1
LIC	leukemia-initiating cells
LKB1	liver kinase B1
LSC	leukemia stem cells
LysoPL	lysophospholipid
MAM	mitochondria-associated membranes
MAPK	mitogen-activated protein kinases
MCP-1	monocyte chemoattractant protein-1
MDM2	mouse double minute 2, an oncogene
MMP	mitochondrial membrane potential
MMPs	matrix metalloproteinases
mPTP	mitochondrial permeability transition pore
MSC	mesenchymal stem cells
mTORC	mammalian target of rapamycin complexes
MTP	mitochondrial trifunctional protein
NADPH	nicotinamide adenine dinucleotide phosphate (reduced)
NF-κB	nuclear factor kappa B
NRF2	Nuclear factor erythroid 2-related factor 2
OMM	outer mitochondrial membrane
OXPHOS	oxidative phosphorylation
PDK1	phosphoinositide-dependent protein kinase 1
PD-L1	programmed cell death 1 ligand
PGC-1α	peroxisome proliferator-activated receptor gamma coactivator 1-alpha
PI3K	phosphoinositide 3-kinase
PIP3	phosphatidylinositol (3,4,5)-trisphosphate
PK	pyruvate kinase
PKM	pyruvate kinase muscle (M) type
PPAR-γ	peroxisome proliferator-activated receptor gamma
PPP	pentose phosphate pathway
PUMA	p53 upregulated modulator of apoptosis
RAS	rat sarcoma pathway
SDF	stromal cell-derived factor
SIRT1	sirtuin molecule 1
SMAD	suppressor of mothers against decapentaplegic, transcription factors
SNAP	synaptosome-associated protein
SNARE	soluble N-ethylmaleimide-sensitive factor attachment protein receptor
STAT	signal transducer and activator of transcription
TAA	tumor-associated adipocytes
TCA	tricarboxylic acid cycle
TGFβ	transforming growth factor beta
TIGAR	TP53-induced glycolysis and apoptosis regulator
TKI	tyrosine kinase inhibitors
TNF-α	tumor necrosis factor alpha

## References

[1] Tortorella S.M., Hung A., Karagiannis T.C. (2015). The implication of cancer progenitor cells and the role of epigenetics in the development of novel therapeutic strategies for chronic myeloid leukemia. Antioxid. Redox Signal..

[2] Hamilton A., Helgason G.V., Schemionek M., Zhang B., Myssina S., Allan E.K., Nicolini F.E., Müller-Tidow C., Bhatia R., Brunton V.G. (2012). Chronic myeloid leukemia stem cells are not dependent on Bcr-Abl kinase activity for their survival. Blood.

[3] de Beauchamp L., Himonas E., Helgason G.V. (2022). Mitochondrial metabolism as a potential therapeutic target in myeloid leukaemia. Leukemia.

[4] Poudel G., Tolland M.G., Hughes T.P., Pagani I.S. (2022). Mechanisms of Resistance and Implications for Treatment Strategies in Chronic Myeloid Leukaemia. Cancers.

[5] Pellicano F., Scott M.T., Helgason G.V., Hopcroft L.E., Allan E.K., Aspinall-O’Dea M., Copland M., Pierce A., Huntly B.J., Whetton A.D. (2014). The antiproliferative activity of kinase inhibitors in chronic myeloid leukemia cells is mediated by FOXO transcription factors. Stem Cells.

[6] Yhim H.Y., Lee N.R., Song E.K., Yim C.Y., Jeon S.Y., Lee B., Kim J.A., Kim H.S., Cho E.H., Kwak J.Y. (2016). Long-Term Outcomes after Imatinib Mesylate Discontinuation in Chronic Myeloid Leukemia Patients with Undetectable Minimal Residual Disease. Acta Haematol..

[7] El-Tanani M., Nsairat H., Matalka I.I., Lee Y.F., Rizzo M., Aljabali A.A., Mishra V., Mishra Y., Hromić-Jahjefendić A., Tambuwala M.M. (2024). The impact of the BCR-ABL oncogene in the pathology and treatment of chronic myeloid leukemia. Pathol. Res. Pract..

[8] Agarwal P., Li H., Choi K., Hueneman K., He J., Welner R.S., Starczynowski D.T., Bhatia R. (2021). TNF-α-induced alterations in stromal progenitors enhance leukemic stem cell growth via CXCR2 signaling. Cell Rep..

[9] Camacho V., Kuznetsova V., Welner R.S. (2021). Inflammatory Cytokines Shape an Altered Immune Response During Myeloid Malignancies. Front. Immunol..

[10] Naka K., Ochiai R., Matsubara E., Kondo C., Yang K.M., Hoshii T., Araki M., Araki K., Sotomaru Y., Sasaki K. (2020). The lysophospholipase D enzyme Gdpd3 is required to maintain chronic myelogenous leukaemia stem cells. Nat. Commun..

[11] Gerber J.M., Gucwa J.L., Esopi D., Gurel M., Haffner M.C., Vala M., Nelson W.G., Jones R.J., Yegnasubramanian S. (2013). Genome-wide comparison of the transcriptomes of highly enriched normal and chronic myeloid leukemia stem and progenitor cell populations. Oncotarget.

[12] Naka K. (2021). Role of Lysophospholipid Metabolism in Chronic Myelogenous Leukemia Stem Cells. Cancers.

[13] Zhang B., Li M., McDonald T., Holyoake T.L., Moon R.T., Campana D., Shultz L., Bhatia R. (2013). Microenvironmental protection of CML stem and progenitor cells from tyrosine kinase inhibitors through N-cadherin and Wnt-β-catenin signaling. Blood.

[14] Patterson S.D., Copland M. (2023). The Bone Marrow Immune Microenvironment in CML: Treatment Responses, Treatment-Free Remission, and Therapeutic Vulnerabilities. Curr. Hematol. Malig. Rep..

[15] Ye H., Adane B., Khan N., Sullivan T., Minhajuddin M., Gasparetto M., Stevens B., Pei S., Balys M., Ashton J.M. (2016). Leukemic Stem Cells Evade Chemotherapy by Metabolic Adaptation to an Adipose Tissue Niche. Cell Stem Cell.

[16] Gazi E., Gardner P., Lockyer N.P., Hart C.A., Brown M.D., Clarke N.W. (2007). Direct evidence of lipid translocation between adipocytes and prostate cancer cells with imaging FTIR microspectroscopy. J. Lipid Res..

[17] Jalilivand S., Nabigol M., Bakhtiyaridovvombaygi M., Gharehbaghian A. (2024). Bone marrow mesenchymal stem cell exosomes suppress JAK/STAT signaling pathway in acute myeloid leukemia in vitro. Blood Res..

[18] Raimondo S., Saieva L., Corrado C., Fontana S., Flugy A., Rizzo A., De Leo G., Alessandro R. (2015). Chronic myeloid leukemia-derived exosomes promote tumor growth through an autocrine mechanism. Cell Commun. Signal..

[19] Zhu X., Wang L., Zhang B., Li J., Dou X., Zhao R.C. (2011). TGF-beta1-induced PI3K/Akt/NF-kappaB/MMP9 signalling pathway is activated in Philadelphia chromosome-positive chronic myeloid leukaemia hemangioblasts. J. Biochem..

[20] Mineo M., Garfield S.H., Taverna S., Flugy A., De Leo G., Alessandro R., Kohn E.C. (2011). Exosomes released by K562 chronic myeloid leukemia cells promote angiogenesis in a src-dependent fashion. Angiogenesis.

[21] Duchartre Y., Kim Y.M., Kahn M. (2016). The Wnt signaling pathway in cancer. Crit. Rev. Oncol. Hematol..

[22] Eiring A.M., Kraft I.L., Page B.D., O’Hare T., Gunning P.T., Deininger M.W. (2014). STAT3 as a mediator of BCR-ABL1-independent resistance in chronic myeloid leukemia. Leuk. Suppl..

[23] Loscocco F., Visani G., Galimberti S., Curti A., Isidori A. (2019). BCR-ABL Independent Mechanisms of Resistance in Chronic Myeloid Leukemia. Front. Oncol..

[24] Grassi S., Palumbo S., Mariotti V., Liberati D., Guerrini F., Ciabatti E., Salehzadeh S., Baratè C., Balducci S., Ricci F. (2019). The WNT Pathway Is Relevant for the BCR-ABL1-Independent Resistance in Chronic Myeloid Leukemia. Front. Oncol..

[25] Kuepper M.K., Bütow M., Herrmann O., Ziemons J., Chatain N., Maurer A., Kirschner M., Maié T., Costa I.G., Eschweiler J. (2019). Stem cell persistence in CML is mediated by extrinsically activated JAK1-STAT3 signaling. Leukemia.

[26] Abraham A., Qiu S., Chacko B.K., Li H., Paterson A., He J., Agarwal P., Shah M., Welner R., Darley-Usmar V.M. (2019). SIRT1 regulates metabolism and leukemogenic potential in CML stem cells. J. Clin. Investig..

[27] Sylow L., Long J.Z., Lokurkar I.A., Zeng X., Richter E.A., Spiegelman B.M. (2016). The Cancer Drug Dasatinib Increases PGC-1α in Adipose Tissue but Has Adverse Effects on Glucose Tolerance in Obese Mice. Endocrinology.

[28] Calabretta B., Salomoni P. (2011). Inhibition of autophagy: A new strategy to enhance sensitivity of chronic myeloid leukemia stem cells to tyrosine kinase inhibitors. Leuk. Lymphoma.

[29] Perrotti D., Cesi V., Trotta R., Guerzoni C., Santilli G., Campbell K., Iervolino A., Condorelli F., Gambacorti-Passerini C., Caligiuri M.A. (2002). BCR-ABL suppresses C/EBPalpha expression through inhibitory action of hnRNP E2. Nat. Genet..

[30] Ma D., Liu P., Wang P., Zhou Z., Fang Q., Wang J. (2021). PKC-β/Alox5 axis activation promotes Bcr-Abl-independent TKI-resistance in chronic myeloid leukemia. J. Cell. Physiol..

[31] Zhao C., Blum J., Chen A., Kwon H.Y., Jung S.H., Cook J.M., Lagoo A., Reya T. (2007). Loss of β-catenin impairs the renewal of normal and CML stem cells in vivo. Cancer Cell.

[32] Busch C., Mulholland T., Zagnoni M., Dalby M., Berry C., Wheadon H. (2023). Overcoming BCR::ABL1 dependent and independent survival mechanisms in chronic myeloid leukaemia using a multi-kinase targeting approach. Cell Commun. Signal..

[33] Cea M., Cagnetta A., Cirmena G., Garuti A., Rocco I., Palermo C., Pierri I., Reverberi D., Nencioni A., Ballestrero A. (2013). Tracking molecular relapse of chronic myeloid leukemia by measuring Hedgehog signaling status. Leuk. Lymphoma.

[34] Dierks C., Beigi R., Guo G.R., Zirlik K., Stegert M.R., Manley P., Trussell C., Schmitt-Graeff A., Landwerlin K., Veelken H. (2008). Expansion of Bcr-Abl-positive leukemic stem cells is dependent on Hedgehog pathway activation. Cancer Cell.

[35] Pamuk G.E., Ehrlich L.A. (2024). An Overview of Myeloid Blast-Phase Chronic Myeloid Leukemia. Cancers.

[36] Han Y., Wang Y., Xu Z., Li J., Yang J., Li Y., Shang Y., Luo J. (2013). Effect of bone marrow mesenchymal stem cells from blastic phase chronic myelogenous leukemia on the growth and apoptosis of leukemia cells. Oncol. Rep..

[37] Zhang X., Tu H., Yang Y., Jiang X., Hu X., Luo Q., Li J. (2019). Bone marrow-derived mesenchymal stromal cells promote resistance to tyrosine kinase inhibitors in chronic myeloid leukemia via the IL-7/JAK1/STAT5 pathway. J. Biol. Chem..

[38] Cilloni D., Saglio G. (2012). Molecular pathways: BCR-ABL. Clin. Cancer Res..

[39] Gutiérrez-Castellanos S., Cruz M., Rabelo L., Godínez R., Reyes-Maldonado E., Riebeling-Navarro C. (2004). Differences in BCL-X(L) expression and STAT5 phosphorylation in chronic myeloid leukaemia patients. Eur. J. Haematol..

[40] Fontana F., Giannitti G., Marchesi S., Limonta P. (2024). The PI3K/Akt Pathway and Glucose Metabolism: A Dangerous Liaison in Cancer. Int. J. Biol. Sci..

[41] Shinohara H., Taniguchi K., Kumazaki M., Yamada N., Ito Y., Otsuki Y., Uno B., Hayakawa F., Minami Y., Naoe T. (2015). Anti-cancer fatty-acid derivative induces autophagic cell death through modulation of PKM isoform expression profile mediated by bcr-abl in chronic myeloid leukemia. Cancer Lett..

[42] Ciscato F., Filadi R., Masgras I., Pizzi M., Marin O., Damiano N., Pizzo P., Gori A., Frezzato F., Chiara F. (2020). Hexokinase 2 displacement from mitochondria-associated membranes prompts Ca2+ -dependent death of cancer cells. EMBO Rep..

[43] Cieri D., Vicario M., Giacomello M., Vallese F., Filadi R., Wagner T., Pozzan T., Pizzo P., Scorrano L., Brini M. (2018). SPLICS: A split green fluorescent protein-based contact site sensor for narrow and wide heterotypic organelle juxtaposition. Cell Death Differ..

[44] Tan V.P., Miyamoto S. (2015). HK2/hexokinase-II integrates glycolysis and autophagy to confer cellular protection. Autophagy.

[45] Liang J., Cao R., Wang X., Zhang Y., Wang P., Gao H., Li C., Yang F., Zeng R., Wei P. (2017). Mitochondrial PKM2 regulates oxidative stress-induced apoptosis by stabilizing Bcl2. Cell Res..

[46] Wingelhofer B., Neubauer H.A., Valent P., Han X., Constantinescu S.N., Gunning P.T., Müller M., Moriggl R. (2018). Implications of STAT3 and STAT5 signaling on gene regulation and chromatin remodeling in hematopoietic cancer. Leukemia.

[47] Kollmann S., Grundschober E., Maurer B., Warsch W., Grausenburger R., Edlinger L., Huuhtanen J., Lagger S., Hennighausen L., Valent P. (2019). Twins with different personalities: STAT5B-but not STAT5A-has a key role in BCR/ABL-induced leukemia. Leukemia.

[48] Singh A.M., Reynolds D., Cliff T., Ohtsuka S., Mattheyses A.L., Sun Y., Menendez L., Kulik M., Dalton S. (2012). Signaling network crosstalk in human pluripotent cells: A Smad2/3-regulated switch that controls the balance between self-renewal and differentiation. Cell Stem Cell.

[49] Roy M., Sarkar R., Mukherjee A., Mukherjee S. (2015). Inhibition of crosstalk between Bcr-Abl and PKC signaling by PEITC, augments imatinib sensitivity in chronic myelogenous leukemia cells. Chem. Biol. Interact..

[50] Naka K., Hoshii T., Muraguchi T., Tadokoro Y., Ooshio T., Kondo Y., Nakao S., Motoyama N., Hirao A. (2010). TGF-β-FOXO signalling maintains leukaemia-initiating cells in chronic myeloid leukaemia. Nature.

[51] Toofan P., Busch C., Morrison H., O’Brien S., Jørgensen H., Copland M., Wheadon H. (2018). Chronic myeloid leukaemia cells require the bone morphogenic protein pathway for cell cycle progression and self-renewal. Cell Death Dis..

[52] Hurtz C., Hatzi K., Cerchietti L., Braig M., Park E., Kim Y.M., Herzog S., Ramezani-Rad P., Jumaa H., Müller M.C. (2011). BCL6-mediated repression of p53 is critical for leukemia stem cell survival in chronic myeloid leukemia. J. Exp. Med..

[53] Fernández de Mattos S., Essafi A., Soeiro I., Pietersen A.M., Birkenkamp K.U., Edwards C.S., Martino A., Nelson B.H., Francis J.M., Jones M.C. (2004). FoxO3a and BCR-ABL regulate cyclin D2 transcription through a STAT5/BCL6-dependent mechanism. Mol. Cell. Biol..

[54] Tanabe A., Sahara H. (2020). The metabolic heterogeneity and flexibility of cancer stem cells. Cancers.

[55] Baquero P., Dawson A., Mukhopadhyay A., Kuntz E.M., Mitchell R., Olivares O., Ianniciello A., Scott M.T., Dunn K., Nicastri M.C. (2019). Targeting quiescent leukemic stem cells using second generation autophagy inhibitors. Leukemia.

[56] Ma J., Wang S., Zhang P., Zheng S., Li X., Li J., Pei H. (2024). Emerging roles for fatty acid oxidation in cancer. Genes Dis..

[57] Carracedo A., Cantley L.C., Pandolfi P.P. (2013). Cancer metabolism: Fatty acid oxidation in the limelight. Nat. Rev. Cancer.

[58] Yadav U.P., Singh T., Kumar P., Sharma P., Kaur H., Sharma S., Singh S., Kumar S., Mehta K. (2020). Metabolic Adaptations in Cancer Stem Cells. Front. Oncol..

[59] Jeon S.-M., Chandel N.S., Hay N. (2012). AMPK regulates NADPH homeostasis to promote tumour cell survival during energy stress. Nature.

[60] Ren Y., Shen H.M. (2019). Critical role of AMPK in redox regulation under glucose starvation. Redox Biol..

[61] Tabe Y., Yamamoto S., Saitoh K., Sekihara K., Monma N., Ikeo K., Mogushi K., Shikami M., Ruvolo V., Ishizawa J. (2017). Bone Marrow Adipocytes Facilitate Fatty Acid Oxidation Activating AMPK and a Transcriptional Network Supporting Survival of Acute Monocytic Leukemia Cells. Cancer Res..

[62] Jin H., Zhang L., He J., Wu M., Jia L., Guo J. (2022). Role of FOXO3a Transcription Factor in the Regulation of Liver Oxidative Injury. Antioxidants.

[63] Luengo A., Li Z., Gui D.Y., Sullivan L.B., Zagorulya M., Do B.T., Ferreira R., Naamati A., Ali A., Lewis C.A. (2021). Increased demand for NAD+ relative to ATP drives aerobic glycolysis. Mol. Cell.

[64] Yuan H., Wang Z., Li L., Zhang H., Modi H., Horne D., Bhatia R., Chen W. (2012). Activation of stress response gene SIRT1 by BCR-ABL promotes leukemogenesis. Blood.

[65] Tasneem A., Sharma A., Syed M.A., Dohare R. (2024). Transcriptomic analysis delineates potential regulatory network as therapeutic alternatives in chronic myeloid leukemia. Egypt. J. Med. Hum. Genet..

[66] Wang Y., Peng J., Yang D., Xing Z., Jiang B., Ding X., Jiang C., Ouyang B., Su L. (2024). From metabolism to malignancy: The multifaceted role of PGC1α in cancer. Front. Oncol..

[67] Duncan M., DeLuca T.A., Kuo H.Y., Yi M., Mrksich M., Miller W.M. (2016). SIRT1 is a critical regulator of K562 cell growth, survival, and differentiation. Exp. Cell Res..

[68] Fang J., Zhou S.-H., Fan J., Yan S.-X. (2015). Roles of glucose transporter-1 and the phosphatidylinositol 3-kinase/protein kinase B pathway in cancer radioresistance. Mol. Med. Rep..

[69] Warsch W., Kollmann K., Eckelhart E., Fajmann S., Cerny-Reiterer S., Hölbl A., Gleixner K.V., Dworzak M., Mayerhofer M., Hoermann G. (2011). High STAT5 levels mediate imatinib resistance and indicate disease progression in chronic myeloid leukemia. Blood.

[70] Walker S.R., Nelson E.A., Yeh J.E., Pinello L., Yuan G.C., Frank D.A. (2013). STAT5 outcompetes STAT3 to regulate the expression of the oncogenic transcriptional modulator BCL6. Mol. Cell. Biol..

[71] Halim C.E., Deng S., Ong M.S., Yap C.T. (2020). Involvement of STAT5 in Oncogenesis. Biomedicines.

[72] Takeuchi A., Nishioka C., Ikezoe T., Yang J., Yokoyama A. (2015). STAT5A regulates DNMT3A in CD34(+)/CD38(-) AML cells. Leuk. Res..

[73] Cheon H., Yang J., Stark G.R. (2011). The functions of signal transducers and activators of transcriptions 1 and 3 as cytokine-inducible proteins. J. Interferon Cytokine Res..

[74] Wang Z., Yuan H., Roth M., Stark J.M., Bhatia R., Chen W.Y. (2013). SIRT1 deacetylase promotes acquisition of genetic mutations for drug resistance in CML cells. Oncogene.

[75] Kuntz E.M., Baquero P., Michie A.M., Dunn K., Tardito S., Holyoake T.L., Helgason G.V., Gottlieb E. (2017). Targeting mitochondrial oxidative phosphorylation eradicates therapy-resistant chronic myeloid leukemia stem cells. Nat. Med..

[76] Gallipoli P., Pellicano F., Morrison H., Laidlaw K., Allan E.K., Bhatia R., Copland M., Jørgensen H.G., Holyoake T.L. (2013). Autocrine TNF-α production supports CML stem and progenitor cell survival and enhances their proliferation. Blood.

[77] Shen N., Liu S., Cui J., Li Q., You Y., Zhong Z., Cheng F., Guo A.Y., Zou P., Yuan G. (2019). Tumor necrosis factor α knockout impaired tumorigenesis in chronic myeloid leukemia cells partly by metabolism modification and miRNA regulation. OncoTargets Ther..

[78] Herrmann O., Kuepper M.K., Bütow M., Costa I.G., Appelmann I., Beier F., Luedde T., Braunschweig T., Koschmieder S., Brümmendorf T.H. (2019). Infliximab therapy together with tyrosine kinase inhibition targets leukemic stem cells in chronic myeloid leukemia. BMC Cancer.

[79] Giustacchini A., Thongjuea S., Barkas N., Woll P.S., Povinelli B.J., Booth C.A.G., Sopp P., Norfo R., Rodriguez-Meira A., Ashley N. (2017). Single-cell transcriptomics uncovers distinct molecular signatures of stem cells in chronic myeloid leukemia. Nat. Med..

[80] Welner R.S., Amabile G., Bararia D., Czibere A., Yang H., Zhang H., Pontes L.L., Ye M., Levantini E., Di Ruscio A. (2015). Treatment of chronic myelogenous leukemia by blocking cytokine alterations found in normal stem and progenitor cells. Cancer Cell.

[81] Dokwal S., Ghalaut V.S., Kulshrestha M.R., Bansal P., Ghalaut P.S., Dokwal S.K. (2015). Prognostic Relevance of Tumor Necrosis Factor Alpha (TNF-α) and Beta 2 Microglobulin (B2M) in Chronic Myeloid Leukemia (CML). Sch. Acad. J. Biosci..

[82] Zhang H.H., Halbleib M., Ahmad F., Manganiello V.C., Greenberg A.S. (2002). Tumor necrosis factor-alpha stimulates lipolysis in differentiated human adipocytes through activation of extracellular signal-related kinase and elevation of intracellular cAMP. Diabetes.

[83] Sharma V.M., Puri V. (2016). Mechanism of TNF-α-induced lipolysis in human adipocytes uncovered. Obesity.

[84] Tan X., Cao Z., Li M., Xu E., Wang J., Xiao Y. (2016). TNF-α downregulates CIDEC via MEK/ERK pathway in human adipocytes. Obesity.

[85] Pavlovsky C., Cordoba B.V., Sanchez M.B., Moiraghi B., Varela A., Custidiano R., Fernandez I., Freitas M.J., Ventriglia M.V., Bendek G. (2023). Elevated plasma levels of IL-6 and MCP-1 selectively identify CML patients who better sustain molecular remission after TKI withdrawal. J. Hematol. Oncol..

[86] Abdel Hammed M.R.A., Ahmed Y.A., Adam E.N., Bakry R., Elnaggar M.G. (2022). sVCAM-1, and TGFβ1 in chronic phase, chronic myeloid leukemia patients treated with tyrosine kinase inhibitors. Egypt. J. Immunol..

[87] Wang C.Z., Zhang Z.Q., Zhang Y., Zheng L.F., Liu Y., Yan A.T., Zhang Y.C., Chang Q.H., Sha S., Xu Z.J. (2023). Comprehensive characterization of TGFB1 across hematological malignancies. Sci. Rep..

[88] Yao H., He S. (2021). Multi-faceted role of cancer-associated adipocytes in the tumor microenvironment (Review). Mol. Med. Rep..

[89] Starling S. (2020). Characterizing bone marrow adipocytes. Nat. Rev. Endocrinol..

[90] Maximus P.S., Al Achkar Z., Hamid P.F., Hasnain S.S., Peralta C.A. (2020). Adipocytokines: Are they the Theory of Everything?. Cytokine.

[91] Vukotić M., Kapor S., Simon F., Cokic V., Santibanez J.F. (2024). Mesenchymal stromal cells in myeloid malignancies: Immunotherapeutic opportunities. Heliyon.

[92] Vianello F., Villanova F., Tisato V., Lymperi S., Ho K.K., Gomes A.R., Marin D., Bonnet D., Apperley J., Lam E.W. (2010). Bone marrow mesenchymal stromal cells non-selectively protect chronic myeloid leukemia cells from imatinib-induced apoptosis via the CXCR4/CXCL12 axis. Haematologica.

[93] Müller L., Tunger A., Wobus M., von Bonin M., Towers R., Bornhäuser M., Dazzi F., Wehner R., Schmitz M. (2021). Immunomodulatory Properties of Mesenchymal Stromal Cells: An Update. Front. Cell Dev. Biol..

[94] Wang X., Hu S., Liu L. (2017). Phosphorylation and acetylation modifications of FOXO3a: Independently or synergistically?. Oncol. Lett..

[95] Brunet A., Sweeney L.B., Sturgill J.F., Chua K.F., Greer P.L., Lin Y., Tran H., Ross S.E., Mostoslavsky R., Cohen H.Y. (2004). Stress-dependent regulation of FOXO transcription factors by the SIRT1 deacetylase. Science.

[96] Boccitto M., Kalb R.G. (2011). Regulation of Foxo-dependent transcription by post-translational modifications. Curr. Drug Targets.

[97] Wagle M., Eiring A.M., Wongchenko M., Lu S., Guan Y., Wang Y., Lackner M., Amler L., Hampton G., Deininger M.W. (2016). A role for FOXO1 in BCR-ABL1-independent tyrosine kinase inhibitor resistance in chronic myeloid leukemia. Leukemia.

[98] Ghaffari S., Jagani Z., Kitidis C., Lodish H.F., Khosravi-Far R. (2003). Cytokines and BCR-ABL mediate suppression of TRAIL-induced apoptosis through inhibition of forkhead FOXO3a transcription factor. Proc. Natl. Acad. Sci. USA.

[99] Zhu H.Q., Gao F.H. (2019). Regulatory Molecules and Corresponding Processes of BCR-ABL Protein Degradation. J. Cancer.

[100] Tsukahara F., Maru Y. (2010). Bag1 directly routes immature BCR-ABL for proteasomal degradation. Blood.

[101] Wu L., Yu J., Chen R., Liu Y., Lou L., Wu Y., Huang L., Fan Y., Gao P., Huang M. (2015). Dual inhibition of Bcr-Abl and Hsp90 by C086 potently inhibits the proliferation of imatinib-resistant CML cells. Clin. Cancer Res..

[102] Yang C., Kang Y.Y., Zhu C.Y., Ma Y., Yu P.H., Yang T., Liu Y.Q., Zhang Z.Y., Suzuki N., Ogra Y. (2025). Heat stress targets and degrades BCR::ABL1 oncoproteins to overcome drug-resistance in Philadelphia chromosome-positive acute lymphoblastic leukemia. Leukemia.

[103] Wang Z., Xu T., Che Y., Ma J.A., Jin R., Mao B., Lai X., Mei K., Zhao H., Yuchi Z. (2026). ATTEC-mediated degradation of BCR-ABL in chronic myeloid leukemia cells. Eur. J. Med. Chem..

[104] Zeng C., Nie D., Wang X., Zhong S., Zeng X., Liu X., Qiu K., Peng X., Zhang W., Chen S. (2024). Combined targeting of GPX4 and BCR-ABL tyrosine kinase selectively compromises BCR-ABL+ leukemia stem cells. Mol. Cancer.

[105] Johnson A.R., Qin Y., Cozzo A.J., Freemerman A.J., Huang M.J., Zhao L., Sampey B.P., Milner J.J., Beck M.A., Damania B. (2016). Metabolic reprogramming through fatty acid transport protein 1 (FATP1) regulates macrophage inflammatory potential and adipose inflammation. Mol. Metab..

[106] Hirao T., Yamaguchi M., Kikuya M., Chibana H., Ito K., Aoki S. (2018). Altered intracellular signaling by imatinib increases the anti-cancer effects of tyrosine kinase inhibitors in chronic myelogenous leukemia cells. Cancer Sci..

[107] Yang B., Wang C., Xie Y., Xu L., Wu X., Wu D. (2018). Monitoring tyrosine kinase inhibitor therapeutic responses with a panel of metabolic biomarkers in chronic myeloid leukemia patients. Cancer Sci..

[108] Liang J., Yu M., Li Y., Zhao L., Wei Q. (2024). Glycogen synthase kinase-3: A potential immunotherapeutic target in tumor microenvironment. Biomed. Pharmacother..

[109] Lei Y., Cai S., Zhang J.K., Ding S.Q., Zhang Z.H., Zhang C.D., Dai D.Q., Li Y.S. (2025). The role and mechanism of fatty acid oxidation in cancer drug resistance. Cell Death Discov..

[110] Karlíková R., Široká J., Friedecký D., Faber E., Hrdá M., Mičová K., Fikarová I., Gardlo A., Janečková H., Vrobel I. (2016). Metabolite Profiling of the Plasma and Leukocytes of Chronic Myeloid Leukemia Patients. J. Proteome Res..

[111] Majewski N., Nogueira V., Robey R.B., Hay N. (2004). Akt inhibits apoptosis downstream of BID cleavage via a glucose-dependent mechanism involving mitochondrial hexokinases. Mol. Cell. Biol..

[112] Pastorino J.G., Shulga N., Hoek J.B. (2002). Mitochondrial binding of hexokinase II inhibits Bax-induced cytochrome c release and apoptosis. J. Biol. Chem..

[113] Cheung E.C., Ludwig R.L., Vousden K.H. (2012). Mitochondrial localization of TIGAR under hypoxia stimulates HK2 and lowers ROS and cell death. Proc. Natl. Acad. Sci. USA.

[114] Oliveira T., Lemos D., Jean L., Kawashima J.M., de Azevedo V.R., Salustiano E.J., Rumjanek V.M., Monteiro R.Q. (2022). Detachment of Hexokinase II from Mitochondria Promotes Collateral Sensitivity in Multidrug Resistant Chronic Myeloid Leukemia Cells. Front. Oncol..

[115] Zahra K., Dey T., Ashish, Mishra S.P., Pandey U. (2020). Pyruvate Kinase M2 and Cancer: The Role of PKM2 in Promoting Tumorigenesis. Front. Oncol..

[116] Gao X., Wang H., Yang J.J., Liu X., Liu Z.R. (2012). Pyruvate kinase M2 regulates gene transcription by acting as a protein kinase. Mol. Cell.

[117] Hsu M.C., Hung W.C. (2018). Pyruvate kinase M2 fuels multiple aspects of cancer cells: From cellular metabolism, transcriptional regulation to extracellular signaling. Mol. Cancer.

[118] Xu D., Liang J., Lin J., Yu C. (2019). PKM2: A Potential Regulator of Rheumatoid Arthritis via Glycolytic and Non-Glycolytic Pathways. Front. Immunol..

[119] Park Y.S., Kim D.J., Koo H., Jang S.H., You Y.-M., Cho J.H., Yang S.-J., Yu E.S., Jung Y., Lee D.C. (2016). AKT-induced PKM2 phosphorylation signals for IGF-1-stimulated cancer cell growth. Oncotarget.

[120] Tong L., Xu N., Zhou X., Huang J., wan-Er W., Chen C., Liang L., Liu Q., Xiaoli L. (2018). PKM2 Mediates Chronic Myeloid Leukemia Imatinib Resistance by Regulating Glycolysis Energy Metabolism. Blood.

[121] Yang G.J., Wu J., Leung C.H., Ma D.L., Chen J. (2021). A review on the emerging roles of pyruvate kinase M2 in anti-leukemia therapy. Int. J. Biol. Macromol..

[122] Hitosugi T., Kang S., Vander Heiden M.G., Chung T.W., Elf S., Lythgoe K., Dong S., Lonial S., Wang X., Chen G.Z. (2009). Tyrosine phosphorylation inhibits PKM2 to promote the Warburg effect and tumor growth. Sci. Signal..

[123] Yang P., Li Z., Wang Y., Zhang L., Wu H., Li Z. (2015). Secreted pyruvate kinase M2 facilitates cell migration via PI3K/Akt and Wnt/β-catenin pathway in colon cancer cells. Biochem. Biophys. Res. Commun..

[124] Shafat M.S., Oellerich T., Mohr S., Robinson S.D., Edwards D.R., Marlein C.R., Piddock R.E., Fenech M., Zaitseva L., Abdul-Aziz A. (2017). Leukemic blasts program bone marrow adipocytes to generate a protumoral microenvironment. Blood.

[125] Chimge N.O., Chen M.H., Nguyen C., Zhao Y., Wu X., Gonzalez P.G., Ogana H., Hurwitz S., Teo J.L., Chen X. (2024). A Deeply Quiescent Subset of CML LSC depend on FAO yet Avoid Deleterious ROS by Suppressing Mitochondrial Complex I. Curr. Mol. Pharmacol..

[126] Flis K., Irvine D., Copland M., Bhatia R., Skorski T. (2012). Chronic myeloid leukemia stem cells display alterations in expression of genes involved in oxidative phosphorylation. Leuk. Lymphoma.

[127] Panuzzo C., Jovanovski A., Pergolizzi B., Pironi L., Stanga S., Fava C., Cilloni D. (2020). Mitochondria: A Galaxy in the Hematopoietic and Leukemic Stem Cell Universe. Int. J. Mol. Sci..

[128] Nemkov T., D’Alessandro A., Reisz J.A. (2019). Metabolic underpinnings of leukemia pathology and treatment. Cancer Rep..

[129] Liu Z., Liu W., Wang W., Ma Y., Wang Y., Drum D.L., Cai J., Blevins H., Lee E., Shah S. (2023). Cpt1a-mediated fatty acid oxidation confers cancer cell resistance to immune-mediated cytolytic killing. Proc. Natl. Acad. Sci. USA.

[130] Li Y.J., Fahrmann J.F., Aftabizadeh M., Zhao Q., Tripathi S.C., Zhang C., Yuan Y., Ann D., Hanash S., Yu H. (2022). Fatty acid oxidation protects cancer cells from apoptosis by increasing mitochondrial membrane lipids. Cell Rep..

[131] Samudio I., Harmancey R., Fiegl M., Kantarjian H., Konopleva M., Korchin B., Kaluarachchi K., Bornmann W., Duvvuri S., Taegtmeyer H. (2010). Pharmacologic inhibition of fatty acid oxidation sensitizes human leukemia cells to apoptosis induction. J. Clin. Investig..

[132] Herroon M.K., Rajagurubandara E., Hardaway A.L., Powell K., Turchick A., Feldmann D., Podgorski I. (2013). Bone marrow adipocytes promote tumor growth in bone via FABP4-dependent mechanisms. Oncotarget.

[133] Key C.C., Bishop A.C., Wang X., Zhao Q., Chen G.Y., Quinn M.A., Zhu X., Zhang Q., Parks J.S. (2020). Human GDPD3 overexpression promotes liver steatosis by increasing lysophosphatidic acid production and fatty acid uptake. J. Lipid Res..

[134] Than A., Cheng Y., Foh L.C., Leow M.K., Lim S.C., Chuah Y.J., Kang Y., Chen P. (2012). Apelin inhibits adipogenesis and lipolysis through distinct molecular pathways. Mol. Cell. Endocrinol..

[135] Bertrand C., Valet P., Castan-Laurell I. (2015). Apelin and energy metabolism. Front. Physiol..

[136] He H., Liu X., Lv L., Liang H., Leng B., Zhao D., Zhang Y., Du Z., Chen X., Li S. (2014). Calcineurin suppresses AMPK-dependent cytoprotective autophagy in cardiomyocytes under oxidative stress. Cell Death Dis..

[137] Jin H.R., Wang J., Wang Z.J., Xi M.J., Xia B.H., Deng K., Yang J.L. (2023). Lipid metabolic reprogramming in tumor microenvironment: From mechanisms to therapeutics. J. Hematol. Oncol..

[138] Rohrig F., Schulze A. (2016). The multifaceted roles of fatty acid synthesis in cancer. Nat. Rev. Cancer.

[139] Shang S., Liu J., Hua F. (2022). Protein acylation: Mechanisms, biological functions and therapeutic targets. Signal Transduct. Target. Ther..

[140] Jung H.E., Shim Y.R., Oh J.E., Oh D.S., Lee H.K. (2019). The autophagy protein Atg5 plays a crucial role in the maintenance and reconstitution ability of hematopoietic stem cells. Immune Netw..

[141] Trefts E., Shaw R.J. (2021). AMPK: Restoring metabolic homeostasis over space and time. Mol. Cell.

[142] Vallianou N.G., Evangelopoulos A., Kazazis C. (2013). Metformin and cancer. Rev. Diabet. Stud..

[143] Vakana E., Altman J.K., Glaser H., Donato N.J., Platanias L.C. (2011). Antileukemic effects of AMPK activators on BCR-ABL-expressing cells. Blood.

[144] Kawaguchi M., Aoki S., Hirao T., Morita M., Ito K. (2016). Autophagy is an important metabolic pathway to determine leukemia cell survival following suppression of the glycolytic pathway. Biochem. Biophys. Res. Commun..

[145] Bellodi C., Lidonnici M.R., Hamilton A., Helgason G.V., Soliera A.R., Ronchetti M., Galavotti S., Young K.W., Selmi T., Yacobi R. (2013). Targeting autophagy potentiates tyrosine kinase inhibitor–induced cell death in Philadelphia chromosome–positive cells, including primary CML stem cells. J. Clin. Investig..

[146] Kausar M.A., Anwar S., Khan Y.S., Saleh A.A., Ahmed M.A.A., Kaur S., Iqbal N., Siddiqui W.A., Najm M.Z. (2025). Autophagy and Cancer: Insights into Molecular Mechanisms and Therapeutic Approaches for Chronic Myeloid Leukemia. Biomolecules.

[147] Huang X., Li Y., Shou L., Li L., Chen Z., Ye X., Qian W. (2019). The molecular mechanisms underlying BCR/ABL degradation in chronic myeloid leukemia cells promoted by Beclin1-mediated autophagy. Cancer Manag. Res..

[148] Helgason G.V., Mukhopadhyay A., Karvela M., Salomoni P., Calabretta B., Holyoake T.L. (2013). Autophagy in chronic myeloid leukaemia: Stem cell survival and implication in therapy. Curr. Cancer Drug Targets.

[149] Saito Y., Chapple Richard H., Lin A., Kitano A., Nakada D. (2015). AMPK protects leukemia-initiating cells in myeloid leukemias from metabolic stress in the bone marrow. Cell Stem Cell.

[150] Maggi F., Morelli M.B., Aguzzi C., Zeppa L., Nabissi M., Polidori C., Santoni G., Amantini C. (2023). Calcium influx, oxidative stress, and apoptosis induced by TRPV1 in chronic myeloid leukemia cells: Synergistic effects with imatinib. Front. Mol. Biosci..

[151] Mitchell R., Hopcroft L.E.M., Baquero P., Allan E.K., Hewit K., James D., Hamilton G., Mukhopadhyay A., O’Prey J., Hair A. (2018). Targeting BCR-ABL-Independent TKI Resistance in Chronic Myeloid Leukemia by mTOR and Autophagy Inhibition. J. Natl. Cancer Inst..

[152] Li M., Li J., Zhang S., Zhou L., Zhu Y., Li S., Li Q., Wang J., Song R. (2024). Progress in the study of autophagy-related proteins affecting resistance to chemotherapeutic drugs in leukemia. Front. Cell Dev. Biol..

[153] Ianniciello A., Helgason G.V. (2022). Targeting ULK1 in cancer stem cells: Insight from chronic myeloid leukemia. Autophagy.

[154] Mostazo M.G.C., Kurrle N., Casado M., Fuhrmann D., Alshamleh I., Häupl B., Martín-Sanz P., Brüne B., Serve H., Schwalbe H. (2020). Metabolic Plasticity Is an Essential Requirement of Acquired Tyrosine Kinase Inhibitor Resistance in Chronic Myeloid Leukemia. Cancers.

[155] Greer E.L., Oskoui P.R., Banko M.R., Maniar J.M., Gygi M.P., Gygi S.P., Brunet A. (2007). The energy sensor AMP-activated protein kinase directly regulates the mammalian FOXO3 transcription factor. J. Biol. Chem..

[156] Li X.N., Song J., Zhang L., LeMaire S.A., Hou X., Zhang C., Coselli J.S., Chen L., Wang X.L., Zhang Y. (2009). Activation of the AMPK-FOXO3 pathway reduces fatty acid-induced increase in intracellular reactive oxygen species by upregulating thioredoxin. Diabetes.

[157] Lee K.M., Giltnane J.M., Balko J.M., Schwarz L.J., Guerrero-Zotano A.L., Hutchinson K.E., Nixon M.J., Estrada M.V., Sánchez V., Sanders M.E. (2017). MYC and MCL1 Cooperatively Promote Chemotherapy-Resistant Breast Cancer Stem Cells via Regulation of Mitochondrial Oxidative Phosphorylation. Cell Metab..

[158] Girnun G.D. (2012). The diverse role of the PPARγ coactivator 1 family of transcriptional coactivators in cancer. Semin. Cell Dev. Biol..

[159] Tan Z., Luo X., Xiao L., Tang M., Bode A.M., Dong Z., Cao Y. (2016). The Role of PGC1α in Cancer Metabolism and its Therapeutic Implications. Mol. Cancer Ther..

[160] Duszka K., Gregor A., Guillou H., König J., Wahli W. (2020). Peroxisome Proliferator-Activated Receptors and Caloric Restriction-Common Pathways Affecting Metabolism, Health, and Longevity. Cells.

[161] Abu Shelbayeh O., Arroum T., Morris S., Busch K.B. (2023). PGC-1α Is a Master Regulator of Mitochondrial Lifecycle and ROS Stress Response. Antioxidants.

[162] Degenhardt K., Mathew R., Beaudoin B., Bray K., Anderson D., Chen G., Mukherjee C., Shi Y., Gélinas C., Fan Y. (2006). Autophagy promotes tumor cell survival and restricts necrosis, inflammation, and tumorigenesis. Cancer Cell.

[163] Rabinovich-Nikitin I., Blant A., Dhingra R., Kirshenbaum L.A., Czubryt M.P. (2022). NF-κB p65 Attenuates Cardiomyocyte PGC-1α Expression in Hypoxia. Cells.

[164] Barrès R., Osler M.E., Yan J., Rune A., Fritz T., Caidahl K., Krook A., Zierath J.R. (2009). Non-CpG methylation of the PGC-1alpha promoter through DNMT3B controls mitochondrial density. Cell Metab..

[165] Niso-Santano M., Malik S.A., Pietrocola F., Bravo-San Pedro J.M., Mariño G., Cianfanelli V., Ben-Younès A., Troncoso R., Markaki M., Sica V. (2015). Unsaturated fatty acids induce non-canonical autophagy. EMBO J..

[166] Sun Y., Ren M., Gao G.Q., Gong B., Xin W., Guo H., Zhang X.J., Gao L., Zhao J.J. (2008). Chronic palmitate exposure inhibits AMPKalpha and decreases glucose-stimulated insulin secretion from beta-cells: Modulation by fenofibrate. Acta Pharmacol. Sin..

[167] Sauvat A., Chen G., Müller K., Tong M., Aprahamian F., Durand S., Cerrato G., Bezu L., Leduc M., Franz J. (2018). Trans-Fats Inhibit Autophagy Induced by Saturated Fatty Acids. EBioMedicine.

[168] Zhang M., Chen J., Zhang H., Dong H., Yue Y., Wang S. (2023). Interleukin-10 increases macrophage-mediated chemotherapy resistance via FABP5 signaling in multiple myeloma. Int. Immunopharmacol..

[169] Wan X., Zhu X., Wang H., Feng Y., Zhou W., Liu P., Shen W., Zhang L., Liu L., Li T. (2020). PGC1α protects against hepatic steatosis and insulin resistance via enhancing IL10-mediated anti-inflammatory response. FASEB J..

[170] Yamakuchi M. (2012). MicroRNA Regulation of SIRT1. Front. Physiol..

[171] Do M.T., Kim H.G., Choi J.H., Jeong H.G. (2014). Metformin induces microRNA-34a to downregulate the Sirt1/Pgc-1α/Nrf2 pathway, leading to increased susceptibility of wild-type p53 cancer cells to oxidative stress and therapeutic agents. Free Radic. Biol. Med..

[172] O’Brien C., Ling T., Berman J.M., Culp-Hill R., Reisz J.A., Rondeau V., Jahangiri S., St-Germain J., Macwan V., Astori A. (2023). Simultaneous inhibition of Sirtuin 3 and cholesterol homeostasis targets acute myeloid leukemia stem cells by perturbing fatty acid beta-oxidation and inducing lipotoxicity. Haematologica.

[173] Li H., Xu M., Lee J., He C., Xie Z. (2012). Leucine supplementation increases SIRT1 expression and prevents mitochondrial dysfunction and metabolic disorders in high-fat diet-induced obese mice. Am. J. Physiol. Endocrinol. Metab..

[174] Zhang J., Xiang H., Liu J., Chen Y., He R.R., Liu B. (2020). Mitochondrial Sirtuin 3: New emerging biological function and therapeutic target. Theranostics.

[175] Tseng A.H., Shieh S.S., Wang D.L. (2013). SIRT3 deacetylates FOXO3 to protect mitochondria against oxidative damage. Free Radic. Biol. Med..

[176] Zhao Y., Shen M., Wu L., Yang H., Yao Y., Yang Q., Du J., Liu L., Li Y., Bai Y. (2023). Stromal cells in the tumor microenvironment: Accomplices of tumor progression?. Cell Death Dis..

[177] Corrado C., Saieva L., Raimondo S., Santoro A., De Leo G., Alessandro R. (2016). Chronic myelogenous leukaemia exosomes modulate bone marrow microenvironment through activation of epidermal growth factor receptor. J. Cell. Mol. Med..

[178] Kumar A., Bhattacharyya J., Jaganathan B.G. (2017). Adhesion to stromal cells mediates imatinib resistance in chronic myeloid leukemia through ERK and BMP signaling pathways. Sci. Rep..

[179] Jalilivand S., Izadirad M., Vazifeh Shiran N., Gharehbaghian A., Naserian S. (2024). The effect of bone marrow mesenchymal stromal cell exosomes on acute myeloid leukemia’s biological functions: A focus on the potential role of LncRNAs. Clin. Exp. Med..

[180] Corn K.C., Windham M.A., Rafat M. (2020). Lipids in the tumor microenvironment: From cancer progression to treatment. Prog. Lipid Res..

[181] Guaita-Esteruelas S., Bosquet A., Saavedra P., Gumà J., Girona J., Lam E.W., Amillano K., Borràs J., Masana L. (2017). Exogenous FABP4 increases breast cancer cell proliferation and activates the expression of fatty acid transport proteins. Mol. Carcinog..

[182] Rozovski U., Harris D.M., Li P., Liu Z., Jain P., Ferrajoli A., Burger J., Thompson P., Jain N., Wierda W. (2018). STAT3-activated CD36 facilitates fatty acid uptake in chronic lymphocytic leukemia cells. Oncotarget.

[183] Ahmadian M., Aksu A.M., Dhillon P., Zerbel Z.J., Kelemen Y., Gbayisomore O., Gómez-Banoy N., Chen S.J., Reilly S.M. (2026). Fatty acids promote uncoupled respiration via ATP/ADP carriers in white adipocytes. Nat. Metab..

[184] Strzałka P., Krawiec K., Wiśnik A., Jarych D., Czemerska M., Zawlik I., Pluta A., Wierzbowska A. (2025). The Role of the Sirtuin Family Histone Deacetylases in Acute Myeloid Leukemia—A Promising Road Ahead. Cancers.

[185] Wu H.L., Yang P., Hu W.L., Wang Y.Y., Lu Y.X., Zhang L.C., Fan Y., Xiao H., Li Z. (2016). Overexpression of PKM2 promotes mitochondrial fusion through attenuated p53 stability. Oncotarget.

[186] Wang Y.Y., Attané C., Milhas D., Dirat B., Dauvillier S., Guerard A., Gilhodes J., Lazar I., Alet N., Laurent V. (2017). Mammary adipocytes stimulate breast cancer invasion through metabolic remodeling of tumor cells. JCI Insight.

[187] Deng W., Wang L., Pan M., Zheng J. (2020). The regulatory role of exosomes in leukemia and their clinical significance. J. Int. Med. Res..

[188] Wei Y., Wang D., Jin F., Bian Z., Li L., Liang H., Li M., Shi L., Pan C., Zhu D. (2017). Pyruvate kinase type M2 promotes tumour cell exosome release via phosphorylating synaptosome-associated protein 23. Nat. Commun..

[189] Bonora M., Morganti C., van Gastel N., Ito K., Calura E., Zanolla I., Ferroni L., Zhang Y., Jung Y., Sales G. (2024). A mitochondrial NADPH-cholesterol axis regulates extracellular vesicle biogenesis to support hematopoietic stem cell fate. Cell Stem Cell.

[190] Wilson K.J., Mill C., Lambert S., Buchman J., Wilson T.R., Hernandez-Gordillo V., Gallo R.M., Ades L.M., Settleman J., Riese D.J. (2012). EGFR ligands exhibit functional differences in models of paracrine and autocrine signaling. Growth Factors.

[191] von Joest M., Chen C., Douché T., Chantrel J., Chiche A., Gianetto Q.G., Matondo M., Li H. (2022). Amphiregulin mediates non-cell-autonomous effect of senescence on reprogramming. Cell Rep..

[192] Sayın S., Yıldırım M., Erdoğdu B., Kaplan O., Koç E., Bulduk T., Cömert M., Güney M., Çelebier M., Aylı M. (2025). Metabolomic Profiling and Bioanalysis of Chronic Myeloid Leukemia: Identifying Biomarkers for Treatment Response and Disease Monitoring. Metabolites.

